# Supplementary material for: Functional annotation and Bayesian fine-mapping reveals candidate genes for important agronomic traits in Holstein bulls
Source: Commun Biol. 2019 Jun 18;2:212. doi: 10.1038/s42003-019-0454-y (PMC6582147; doi:10.1038/s42003-019-0454-y)
Supplement: Supplementary file 1 — Supplementary Information [file 42003_2019_454_MOESM1_ESM.pdf]

Supplementary Figures

Supplementary Figure 1. Manhattan plots for 35 dairy traits.

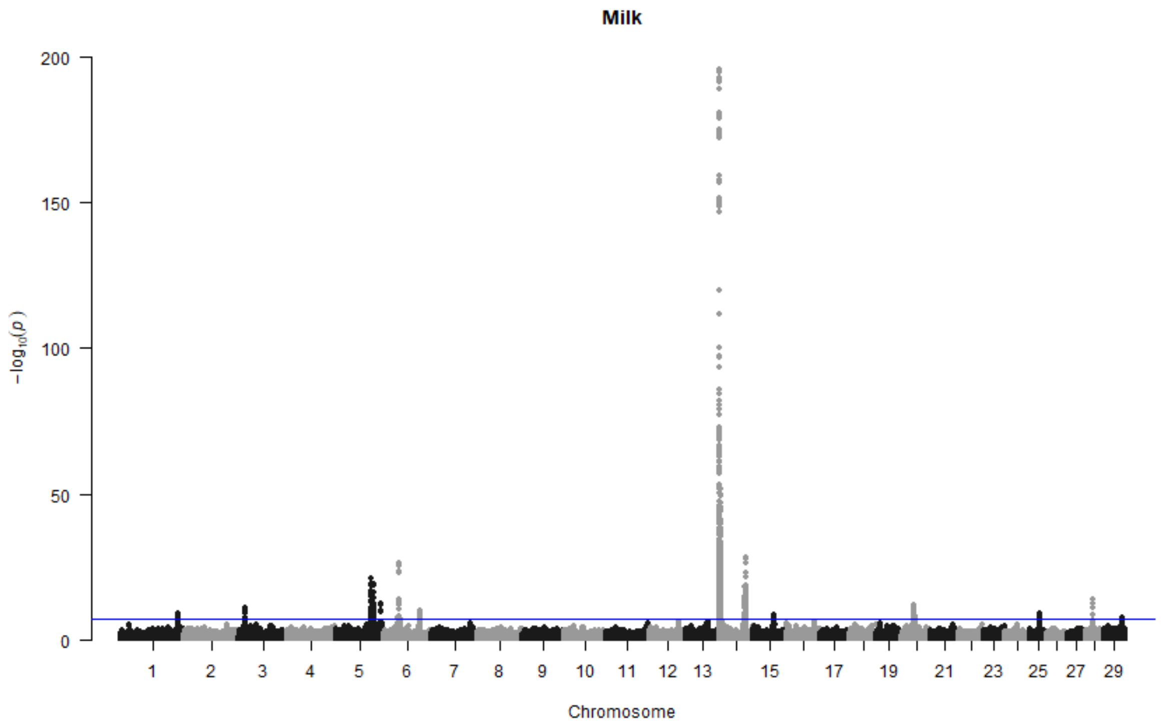

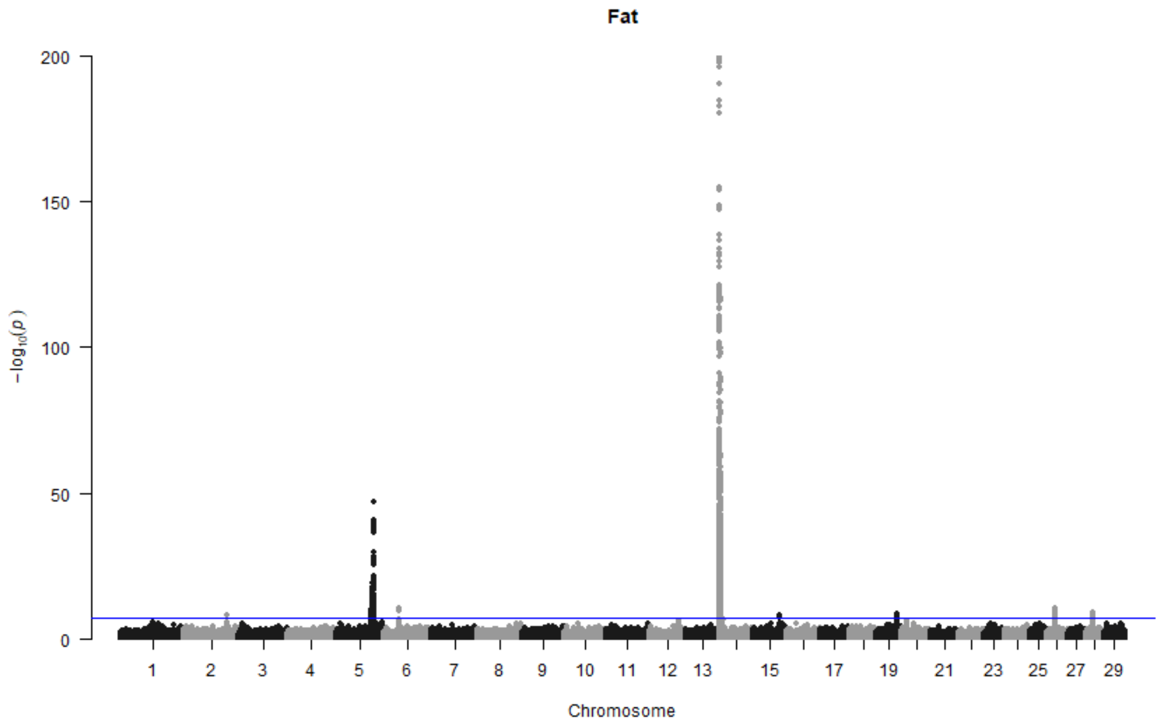

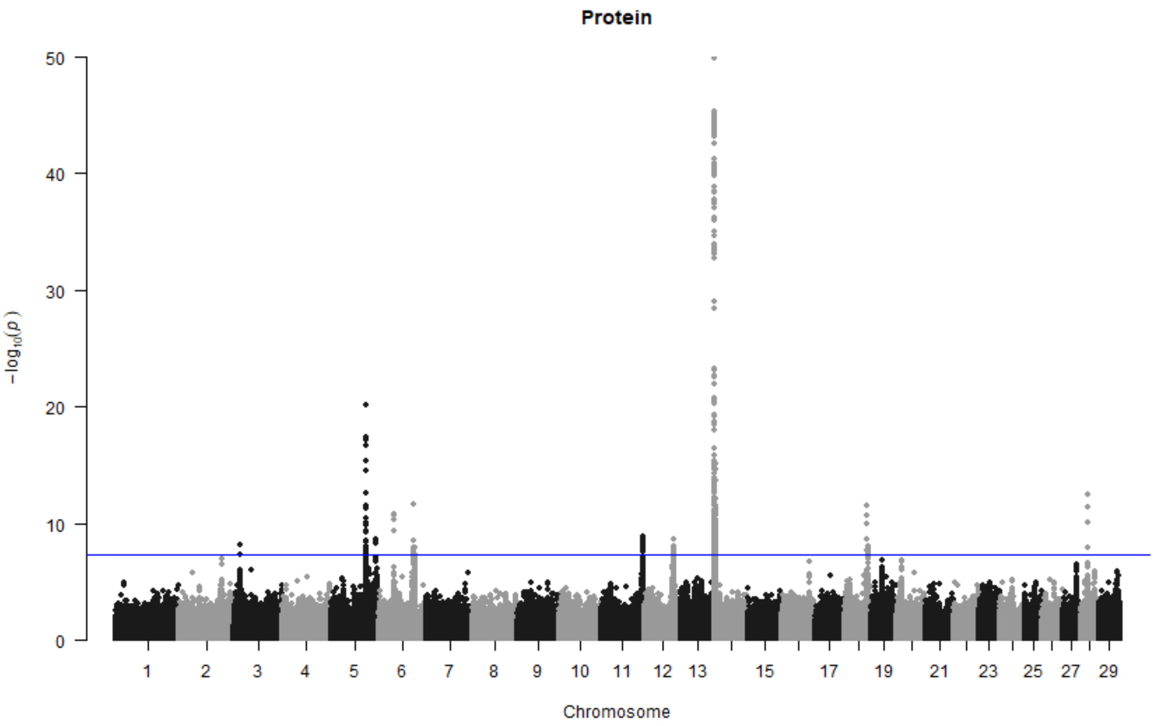
Protein
$-\log_{10}(p)$
0
10
20
30
40
50
1
2
3
4
5
6
7
8
9
10
11
12
13
15
17
19
21
23
25
27
29
Chromosome

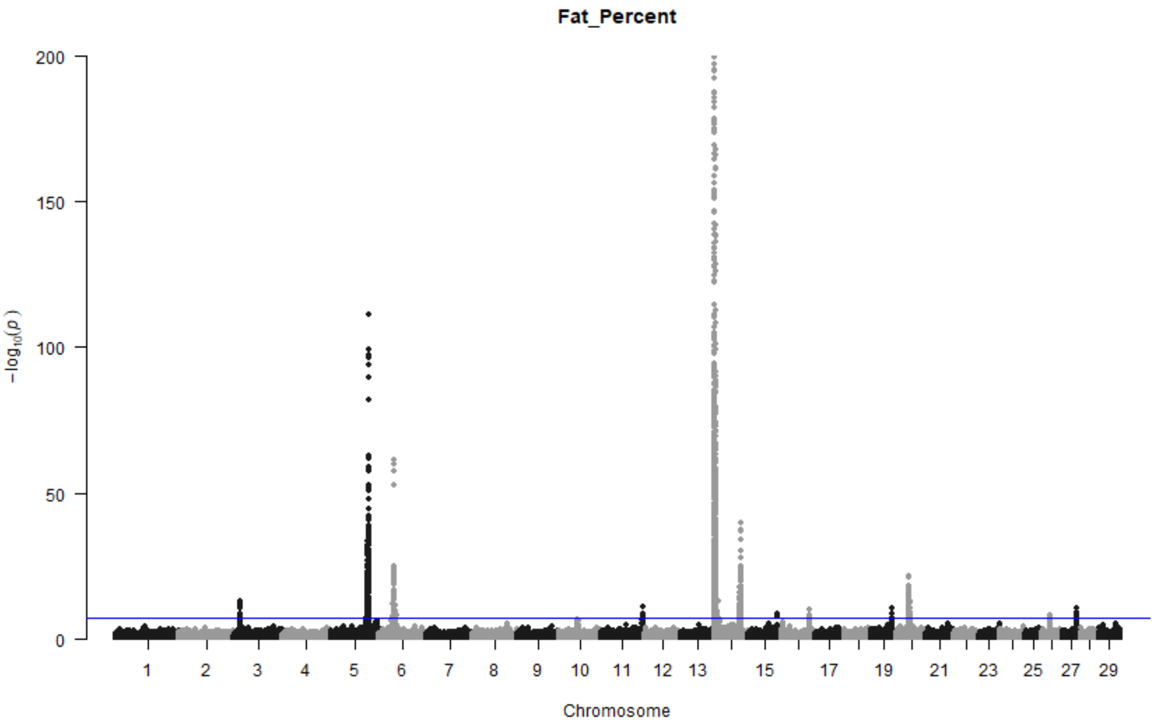
Fat_Percent
$-\log_{10}(p)$
0
50
100
150
200
1
2
3
4
5
6
7
8
9
10
11
12
13
15
17
19
21
23
25
27
29
Chromosome

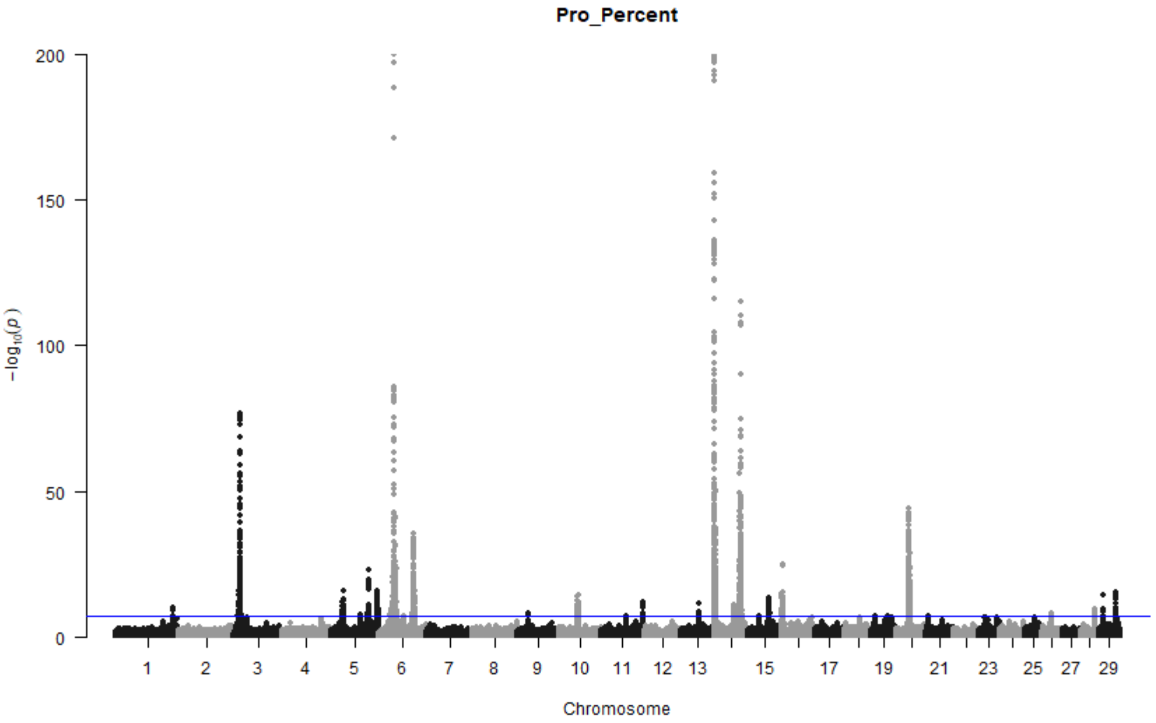
Pro_Percent
200
150
100
50
0
−log10(p)
1
2
3
4
5
6
7
8
9
10
11
12
13
15
17
19
21
23
25
27
29
Chromosome

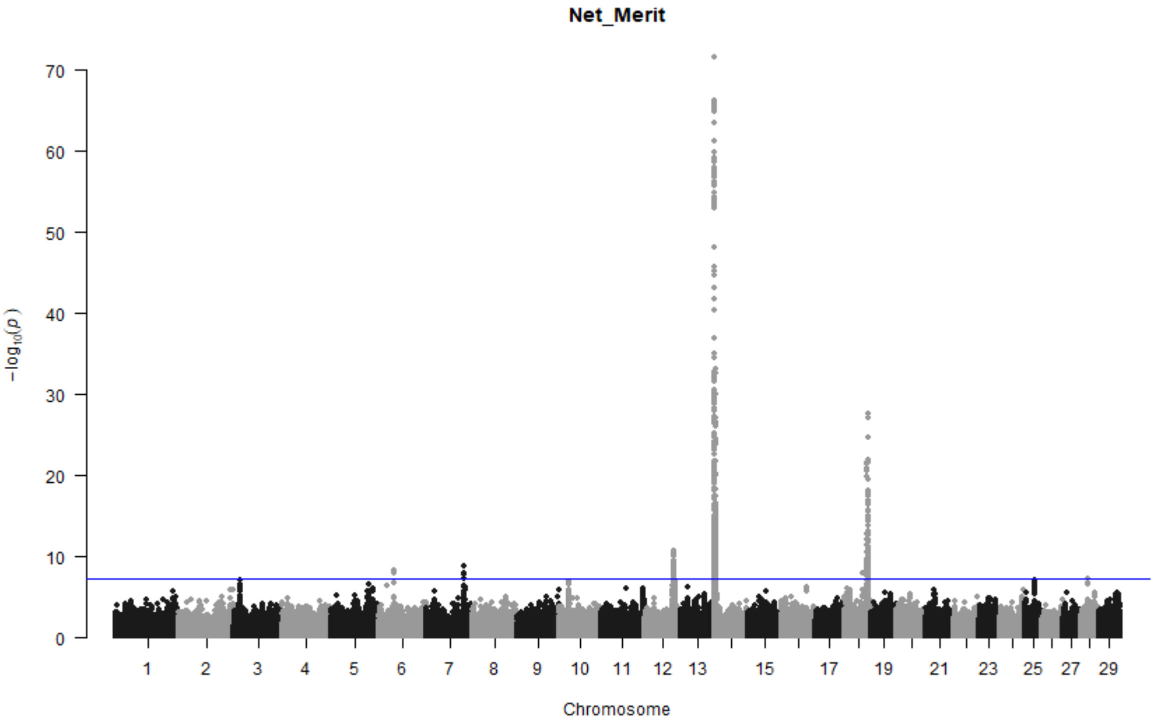
Net_Merit
70
60
50
40
30
20
10
0
−log10(p)
1
2
3
4
5
6
7
8
9
10
11
12
13
15
17
19
21
23
25
27
29
Chromosome

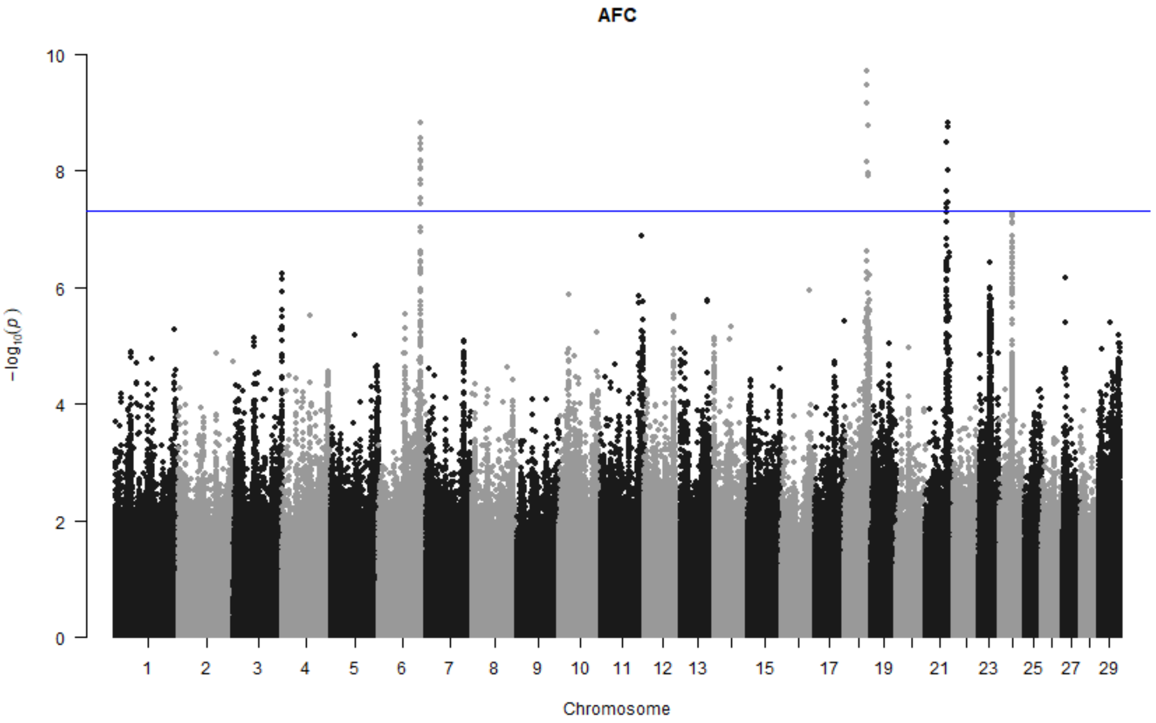
AFC
10
8
6
4
2
0
$-\log_{10}(p)$
1 2 3 4 5 6 7 8 9 10 11 12 13 15 17 19 21 23 25 27 29
Chromosome

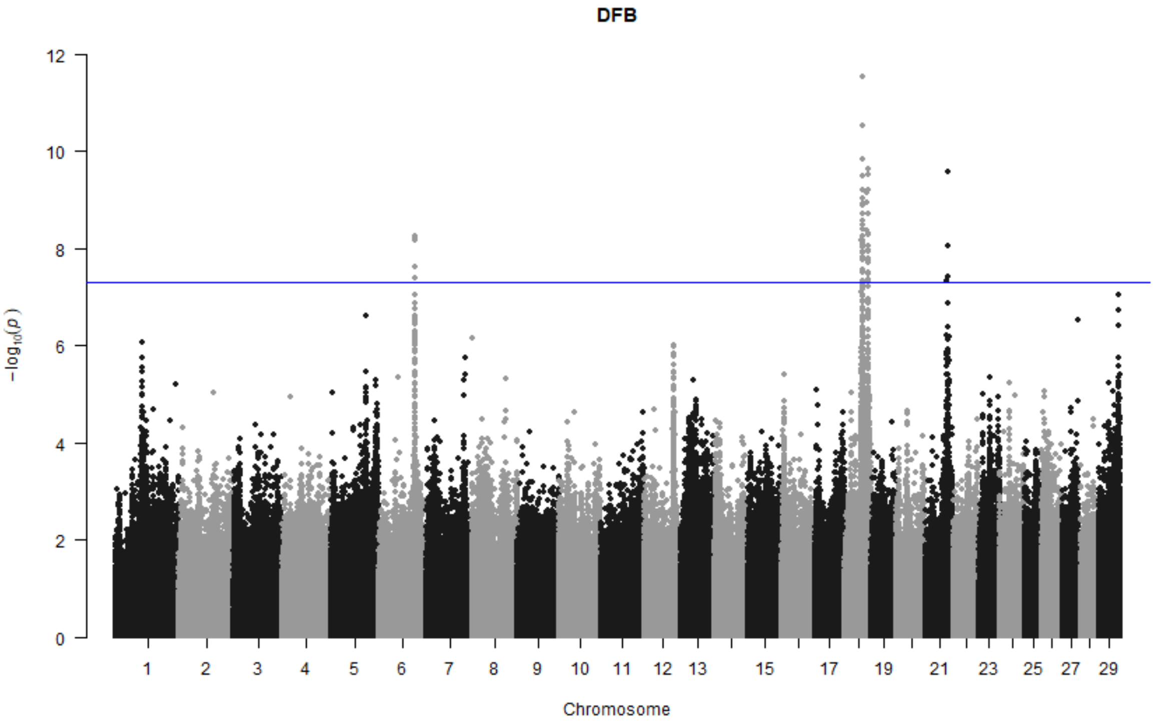
DFB
12
10
8
6
4
2
0
$-\log_{10}(p)$
1 2 3 4 5 6 7 8 9 10 11 12 13 15 17 19 21 23 25 27 29
Chromosome

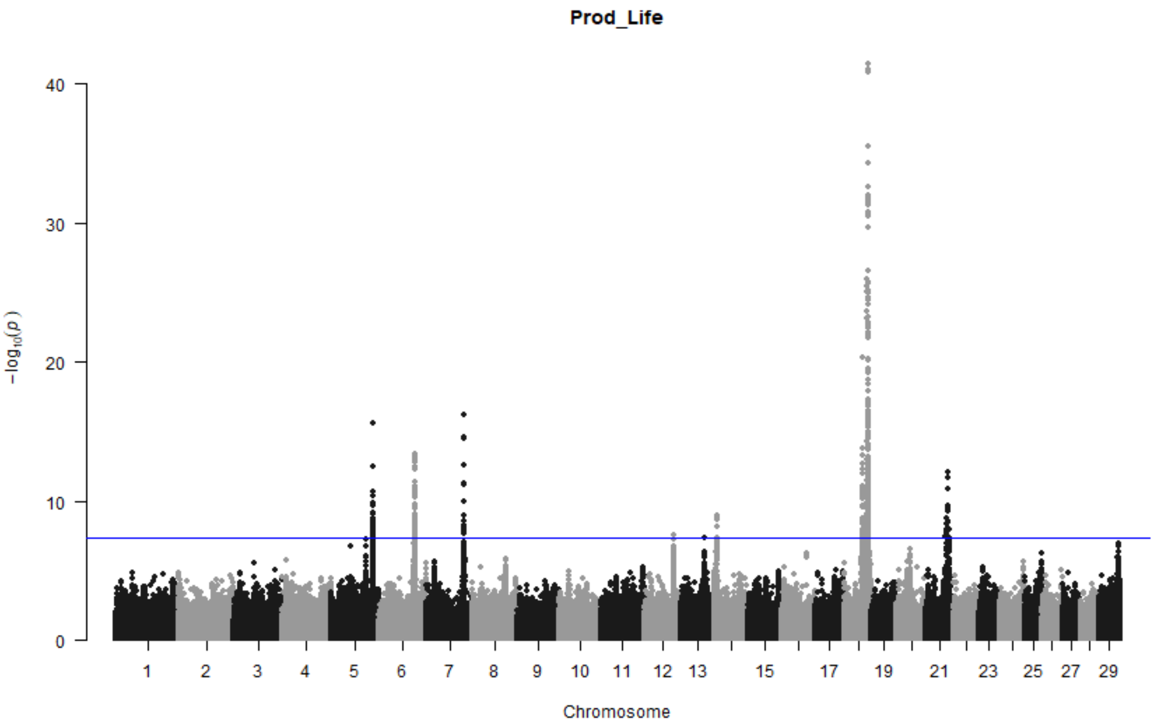

Prod_Life
40
30
20
10
0
−log10(p)
1 2 3 4 5 6 7 8 9 10 11 12 13 15 17 19 21 23 25 27 29
Chromosome

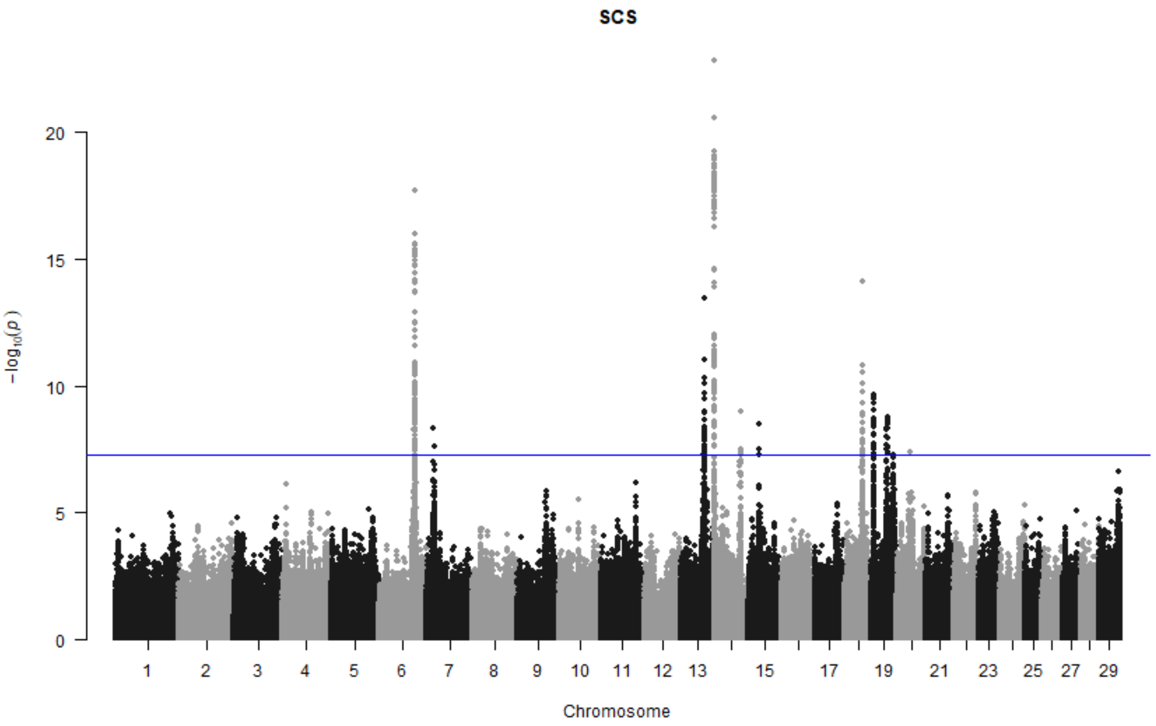

SCS
20
15
10
5
0
−log10(p)
1 2 3 4 5 6 7 8 9 10 11 12 13 15 17 19 21 23 25 27 29
Chromosome

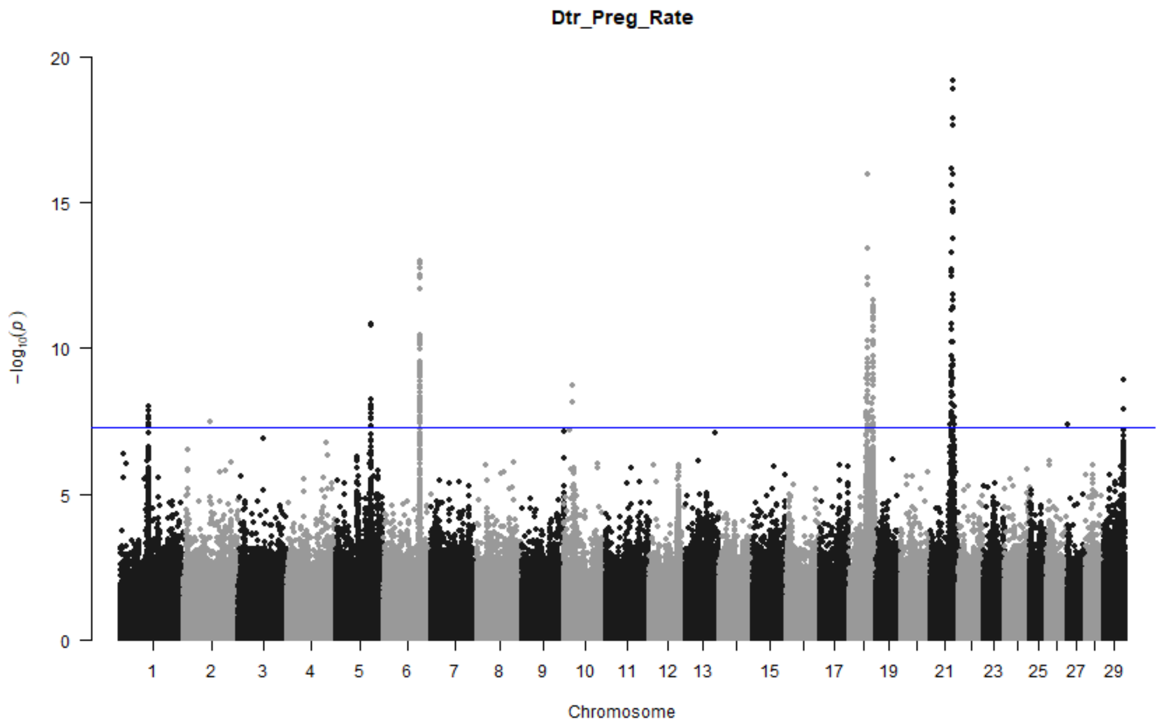
Dtr_Preg_Rate
20
15
10
5
0
−log10(p)
1 2 3 4 5 6 7 8 9 10 11 12 13 15 17 19 21 23 25 27 29
Chromosome

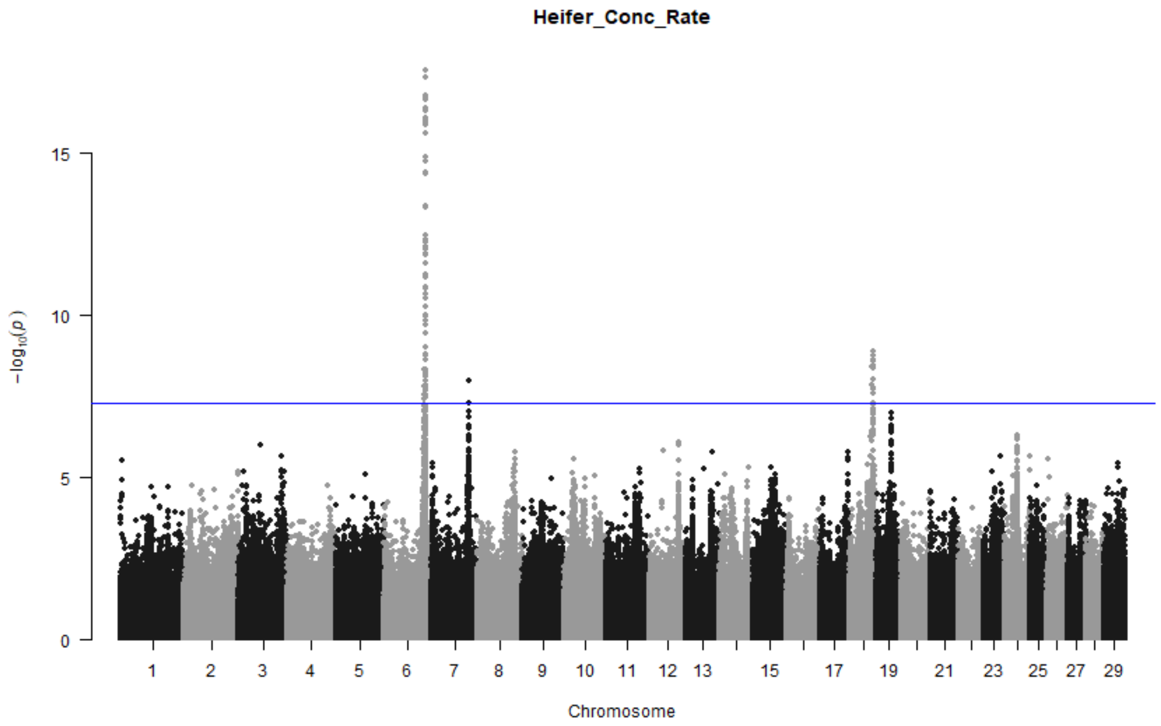
Heifer_Conc_Rate
15
10
5
0
−log10(p)
1 2 3 4 5 6 7 8 9 10 11 12 13 15 17 19 21 23 25 27 29
Chromosome

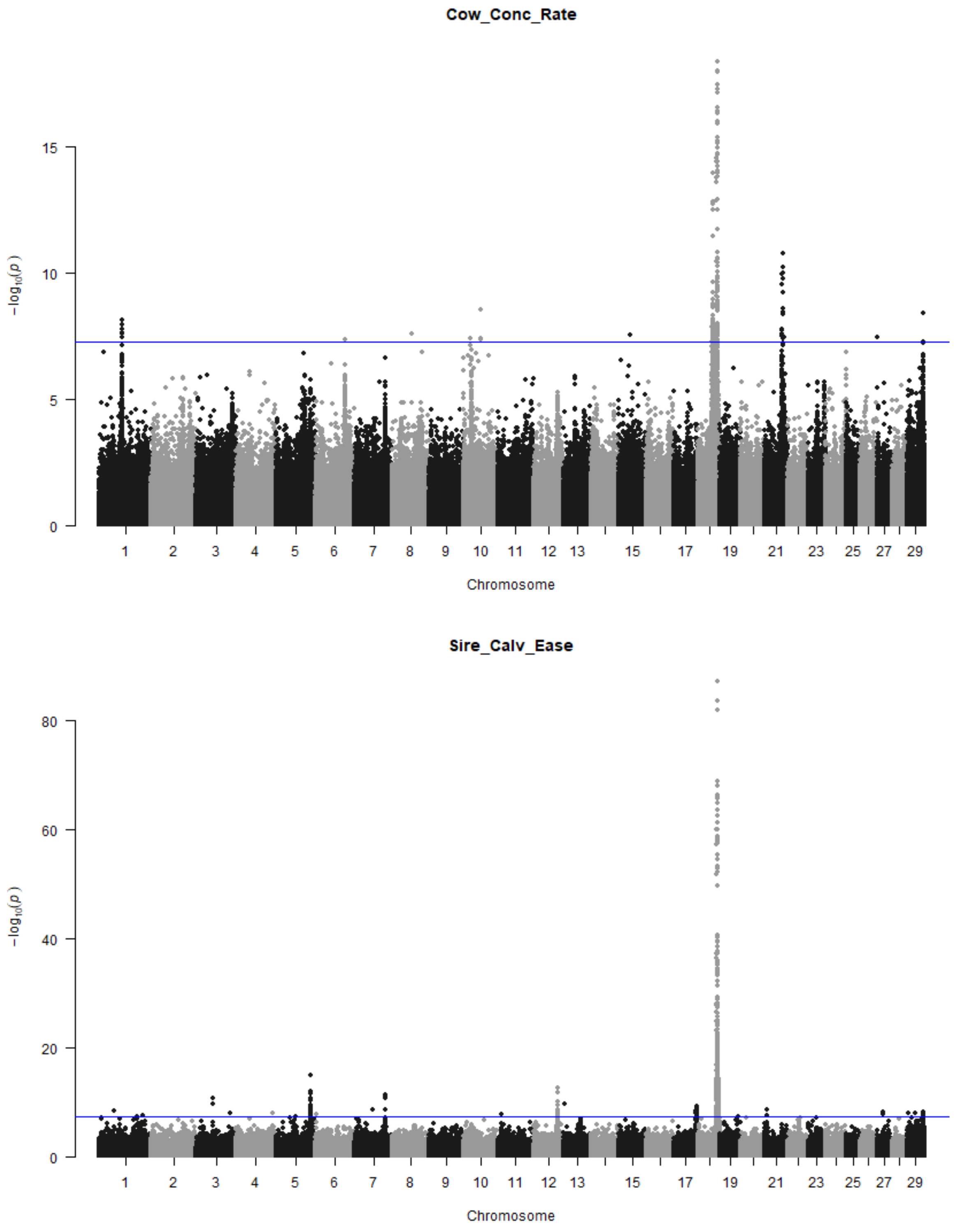

Cow_Conc_Rate
−log10(p)
0
5
10
15
1
2
3
4
5
6
7
8
9
10
11
12
13
15
17
19
21
23
25
27
29
Chromosome
Sire_Calv_Ease
−log10(p)
0
20
40
60
80
1
2
3
4
5
6
7
8
9
10
11
12
13
15
17
19
21
23
25
27
29
Chromosome

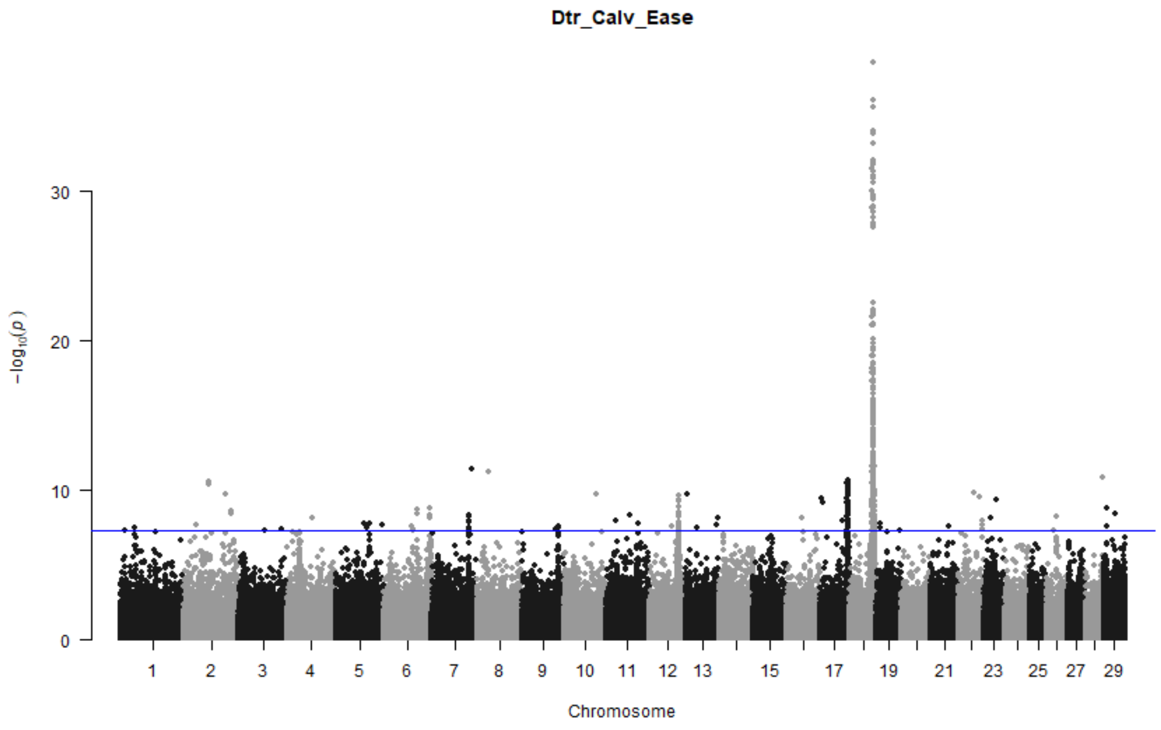
Dtr_Calv_Ease
0
10
20
30
−log10(p)
1
2
3
4
5
6
7
8
9
10
11
12
13
15
17
19
21
23
25
27
29
Chromosome

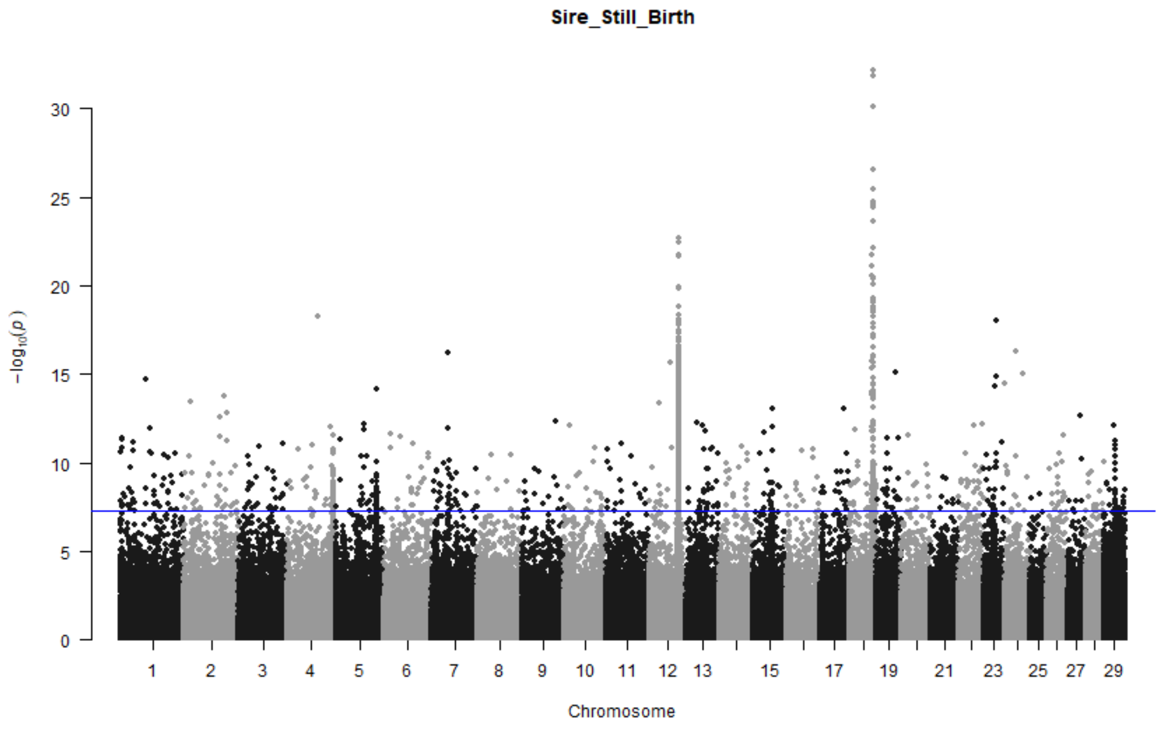
Sire_Still_Birth
0
5
10
15
20
25
30
−log10(p)
1
2
3
4
5
6
7
8
9
10
11
12
13
15
17
19
21
23
25
27
29
Chromosome

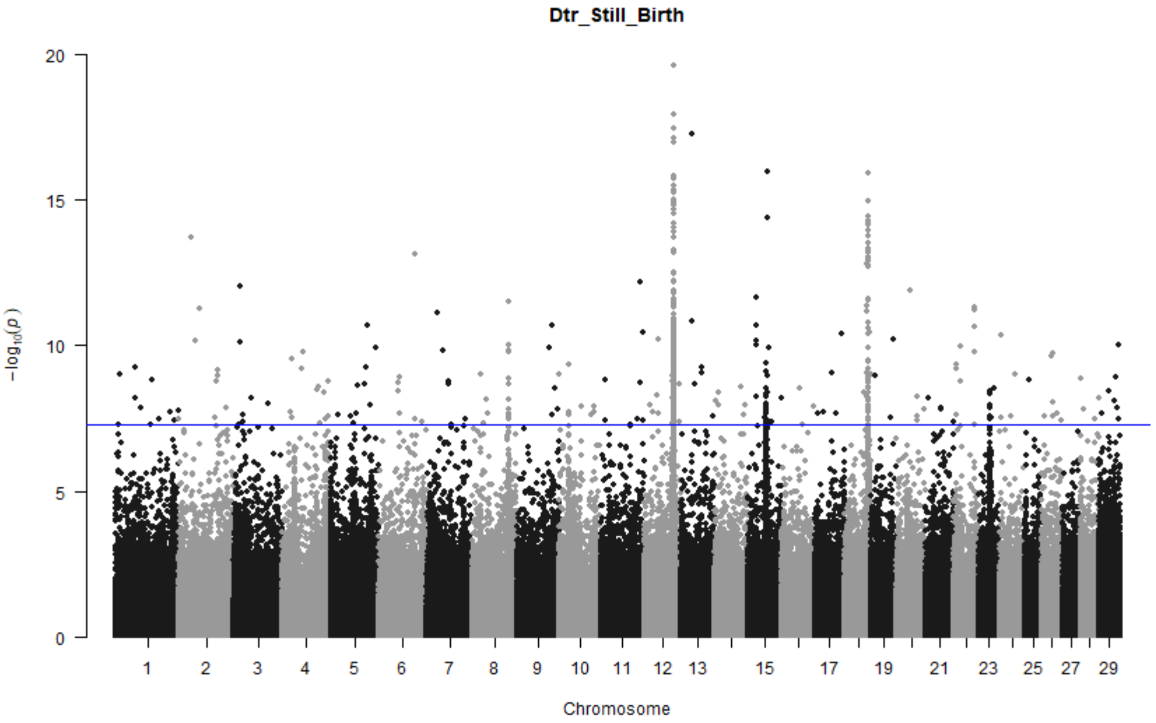

Dtr_Still_Birth
20
15
10
5
0
−log10(p)
1 2 3 4 5 6 7 8 9 10 11 12 13 15 17 19 21 23 25 27 29
Chromosome

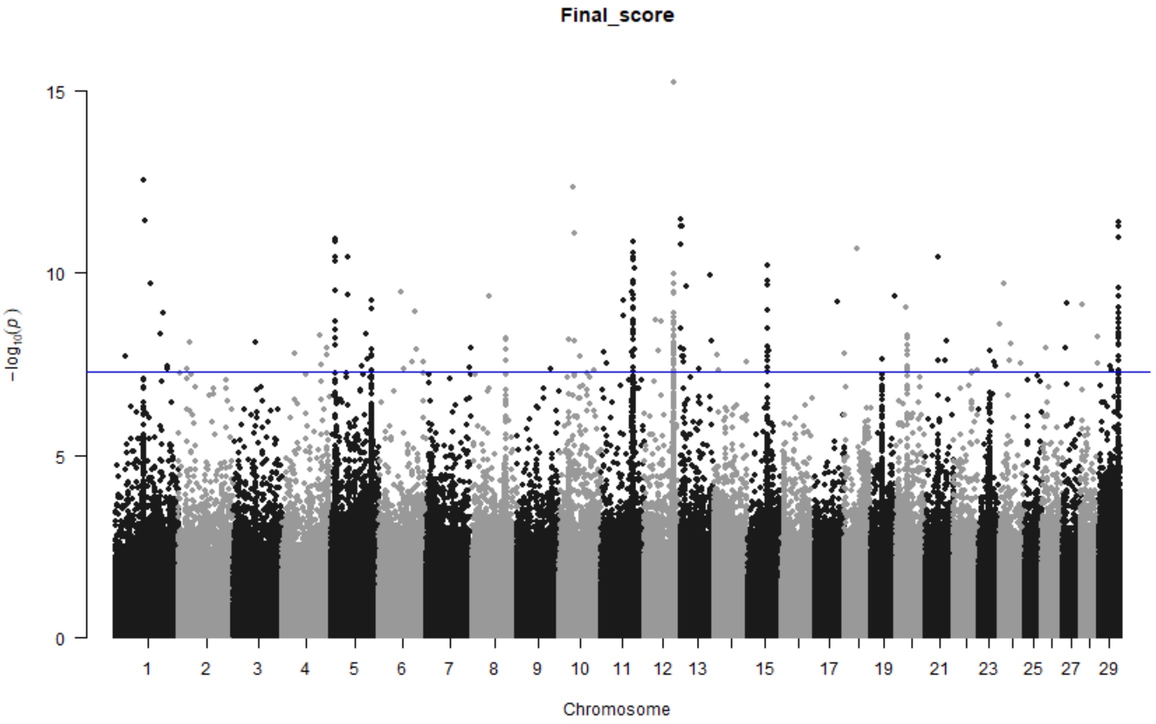

Final_score
15
10
5
0
−log10(p)
1 2 3 4 5 6 7 8 9 10 11 12 13 15 17 19 21 23 25 27 29
Chromosome

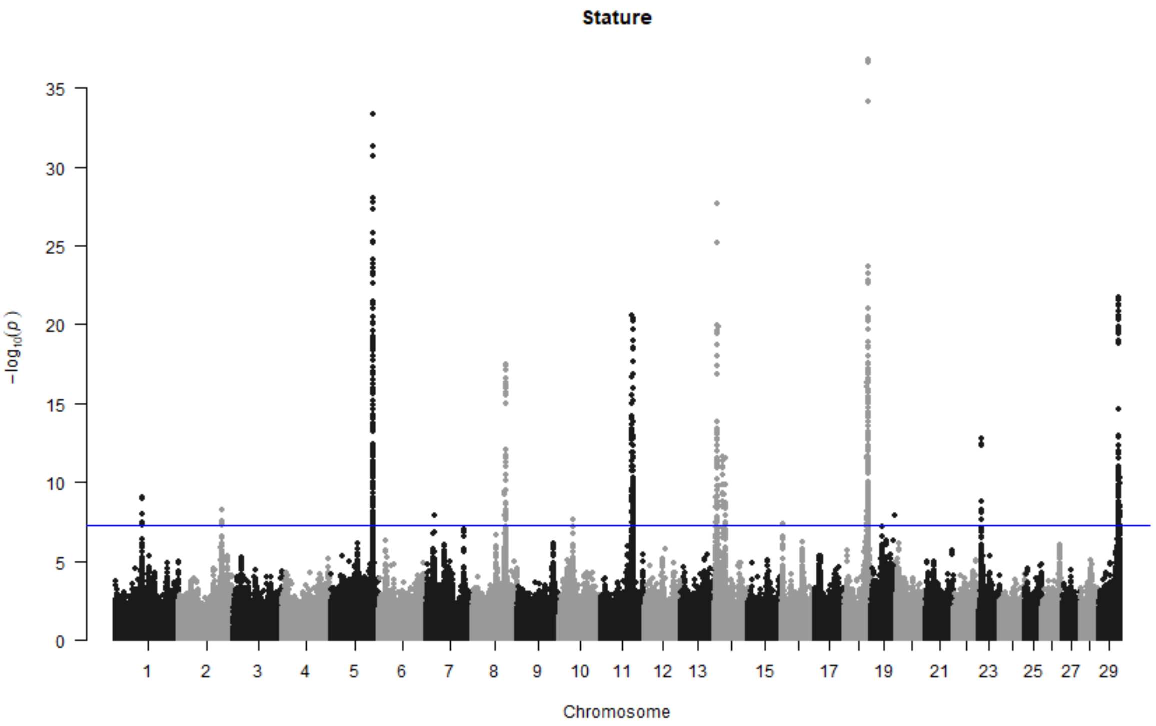
Stature
35
30
25
20
15
10
5
0
−log10(p)
1 2 3 4 5 6 7 8 9 10 11 12 13 15 17 19 21 23 25 27 29
Chromosome

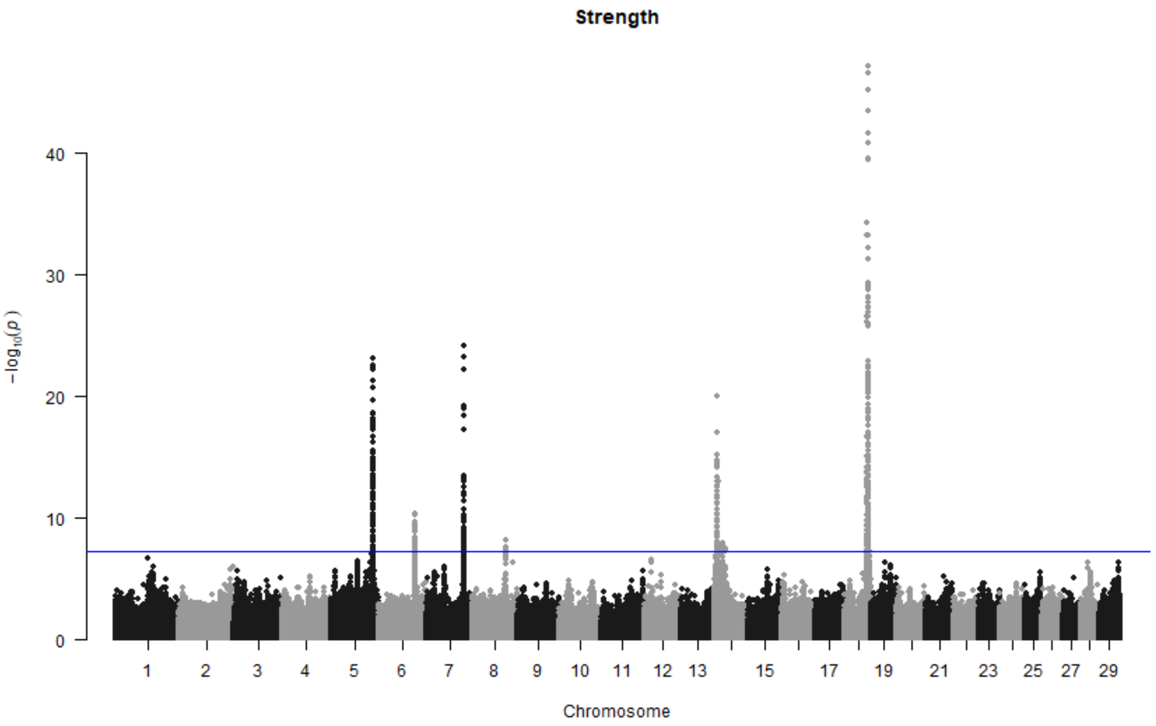
Strength
40
30
20
10
0
−log10(p)
1 2 3 4 5 6 7 8 9 10 11 12 13 15 17 19 21 23 25 27 29
Chromosome

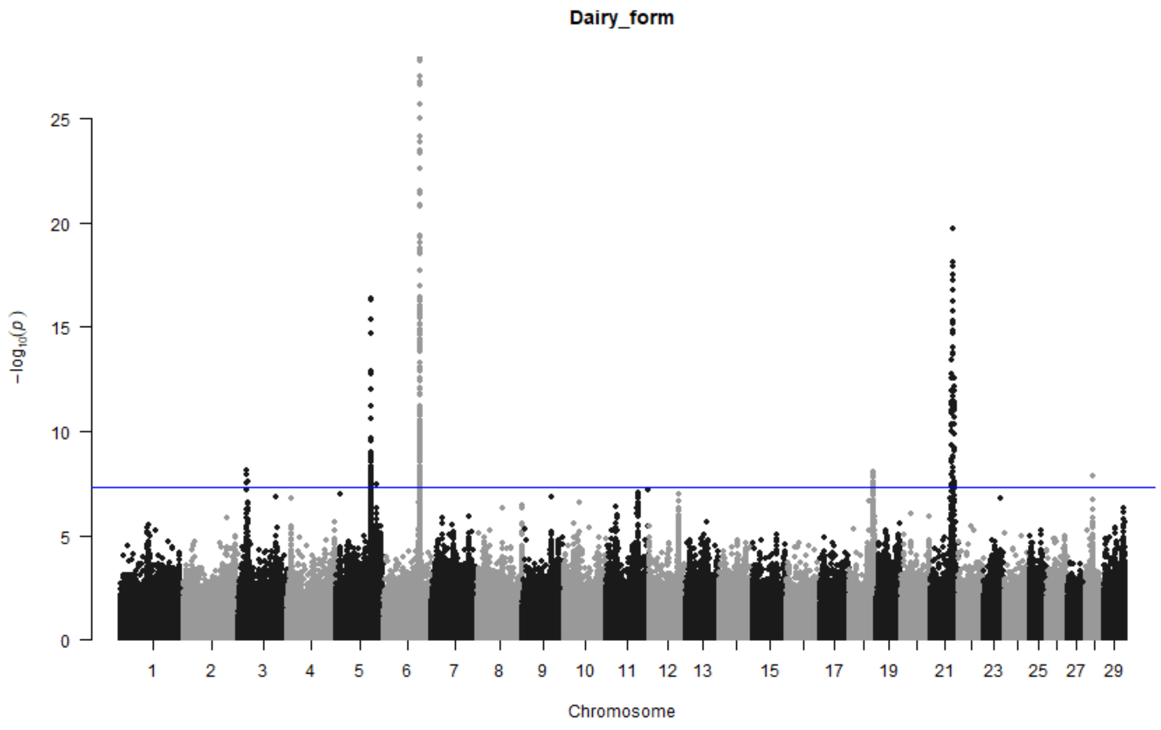
Dairy_form
$-\log_{10}(p)$
0
5
10
15
20
25
1
2
3
4
5
6
7
8
9
10
11
12
13
15
17
19
21
23
25
27
29
Chromosome

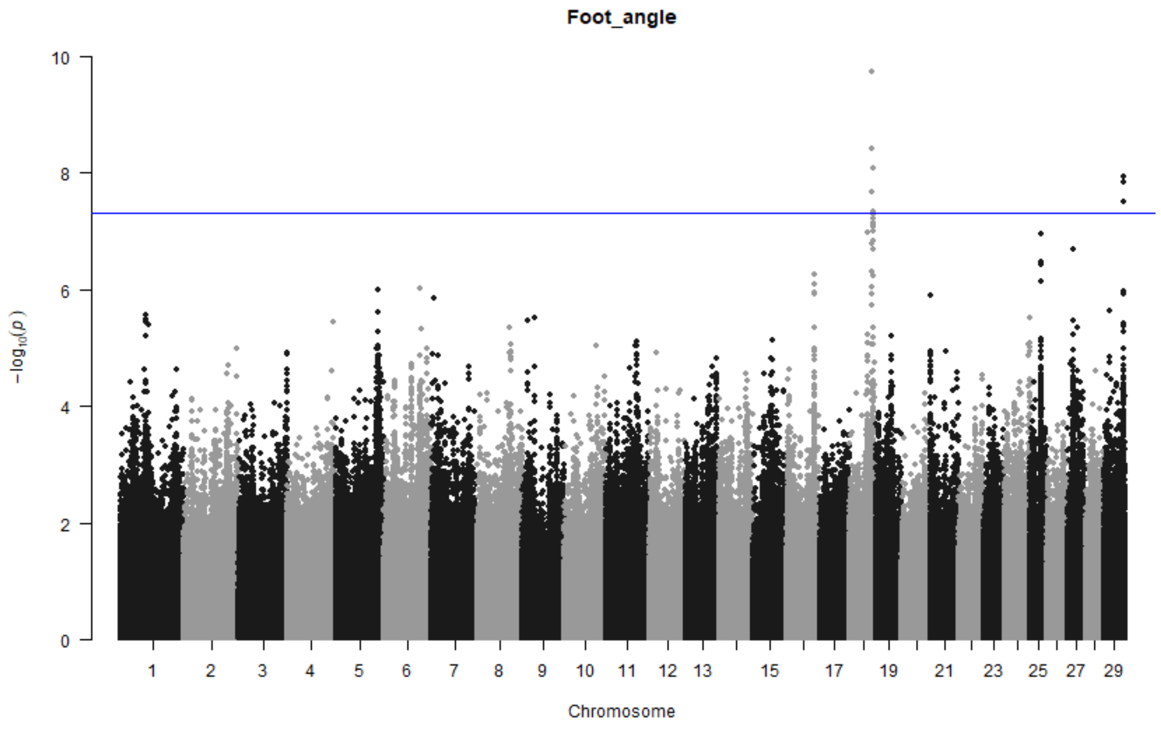
Foot_angle
$-\log_{10}(p)$
0
2
4
6
8
10
1
2
3
4
5
6
7
8
9
10
11
12
13
15
17
19
21
23
25
27
29
Chromosome

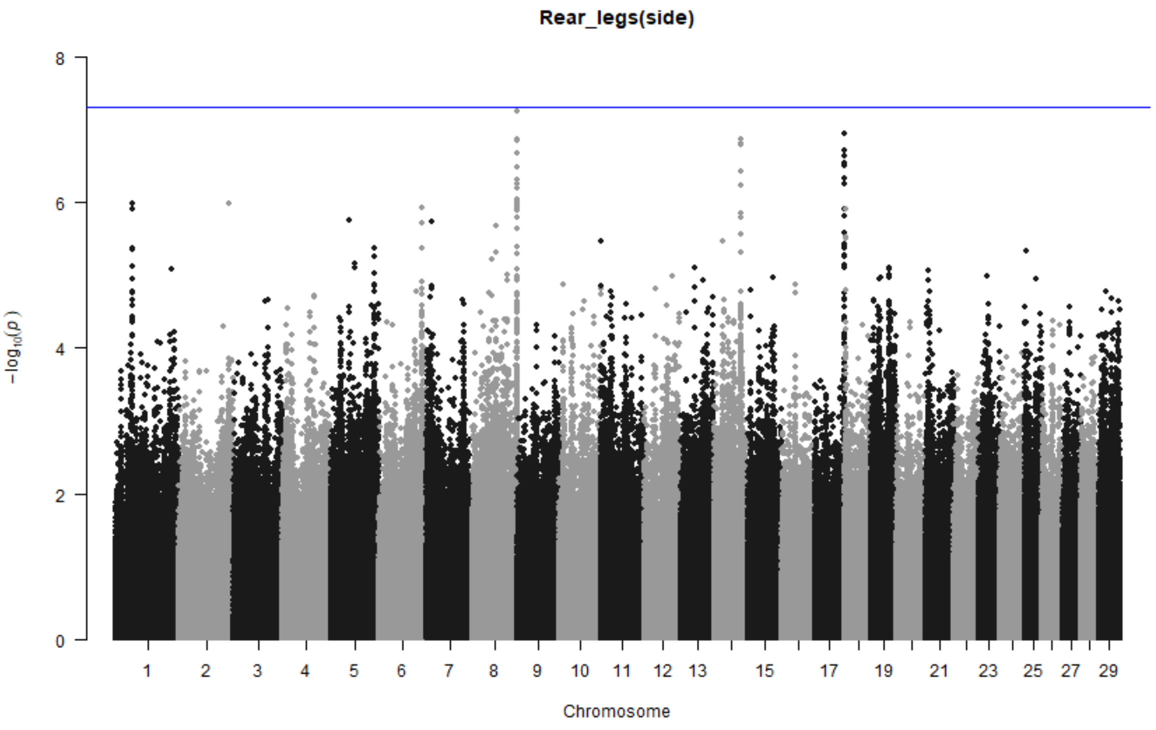
Rear_legs(side)
8
6
4
2
0
−log10(p)
1 2 3 4 5 6 7 8 9 10 11 12 13 15 17 19 21 23 25 27 29
Chromosome

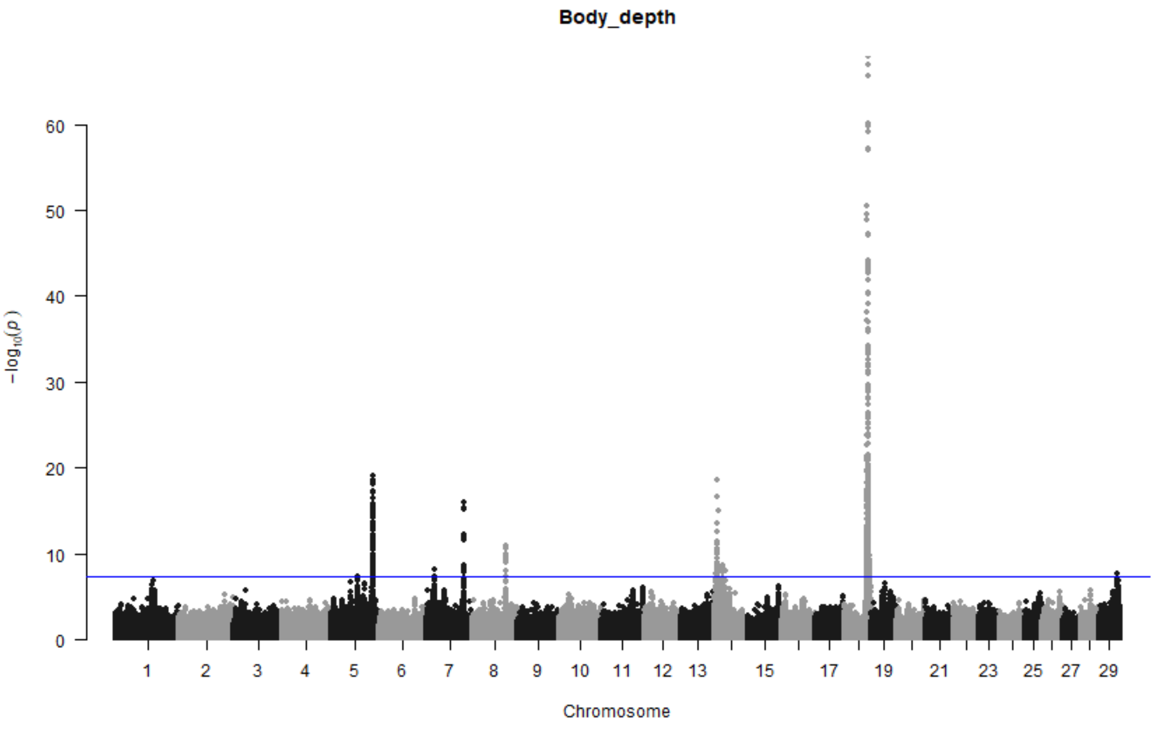
Body_depth
60
50
40
30
20
10
0
−log10(p)
1 2 3 4 5 6 7 8 9 10 11 12 13 15 17 19 21 23 25 27 29
Chromosome

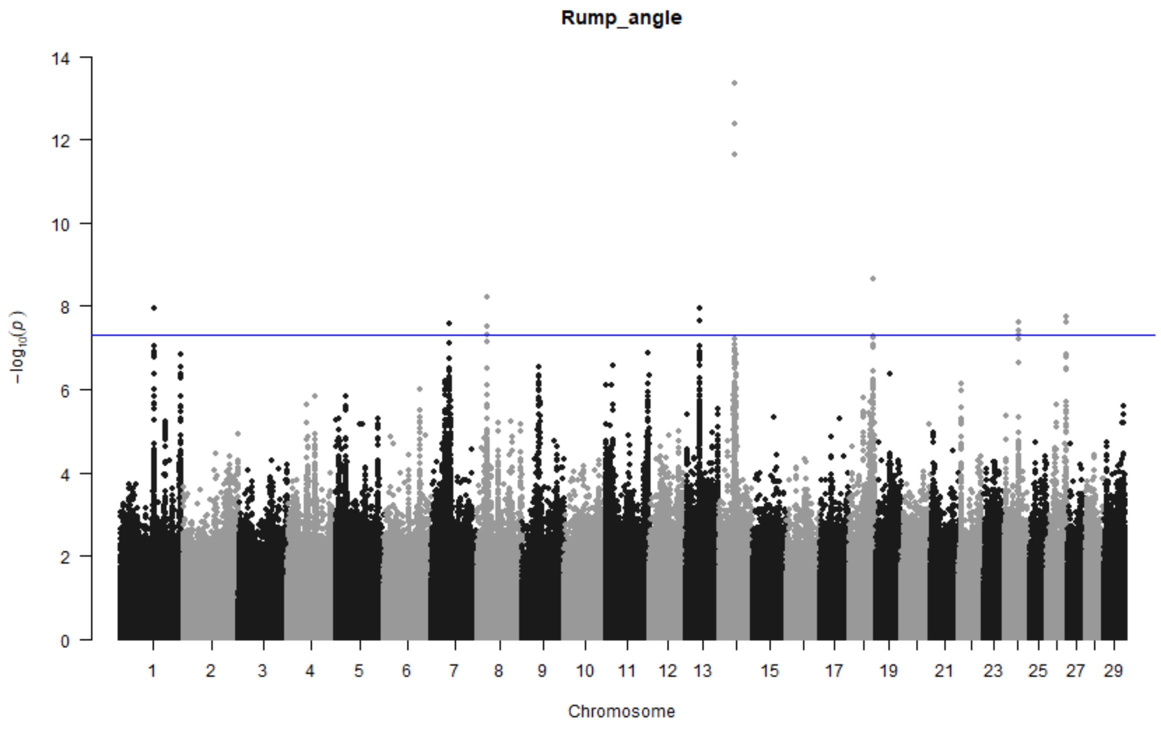

Rump_angle
14
12
10
8
6
4
2
0
−log10(p)
1
2
3
4
5
6
7
8
9
10
11
12
13
15
17
19
21
23
25
27
29
Chromosome

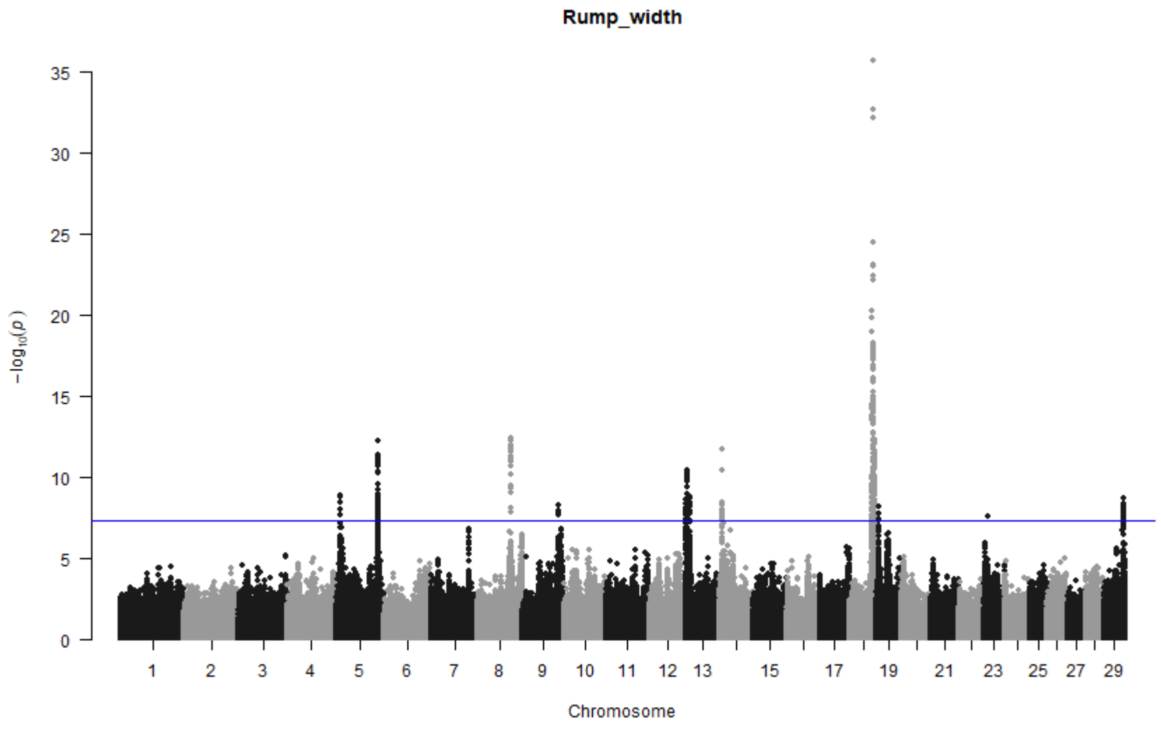

Rump_width
35
30
25
20
15
10
5
0
−log10(p)
1
2
3
4
5
6
7
8
9
10
11
12
13
15
17
19
21
23
25
27
29
Chromosome

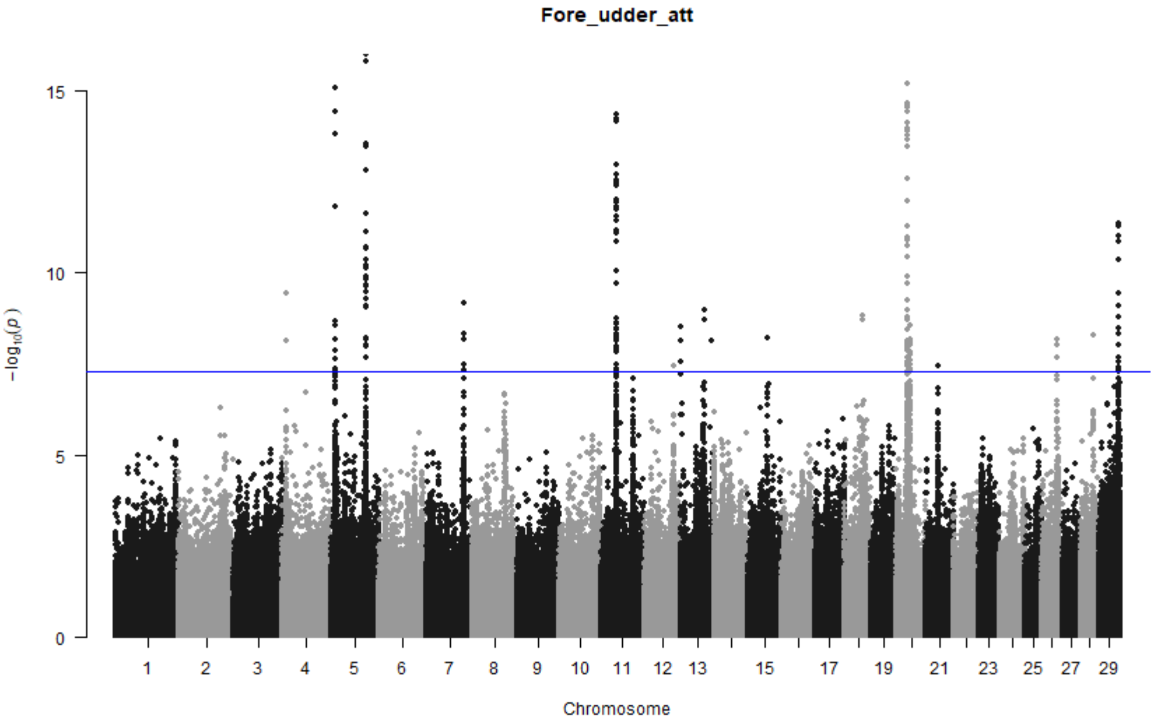
Fore_udder_att
−log10(p)
15
10
5
0
1
2
3
4
5
6
7
8
9
10
11
12
13
15
17
19
21
23
25
27
29
Chromosome

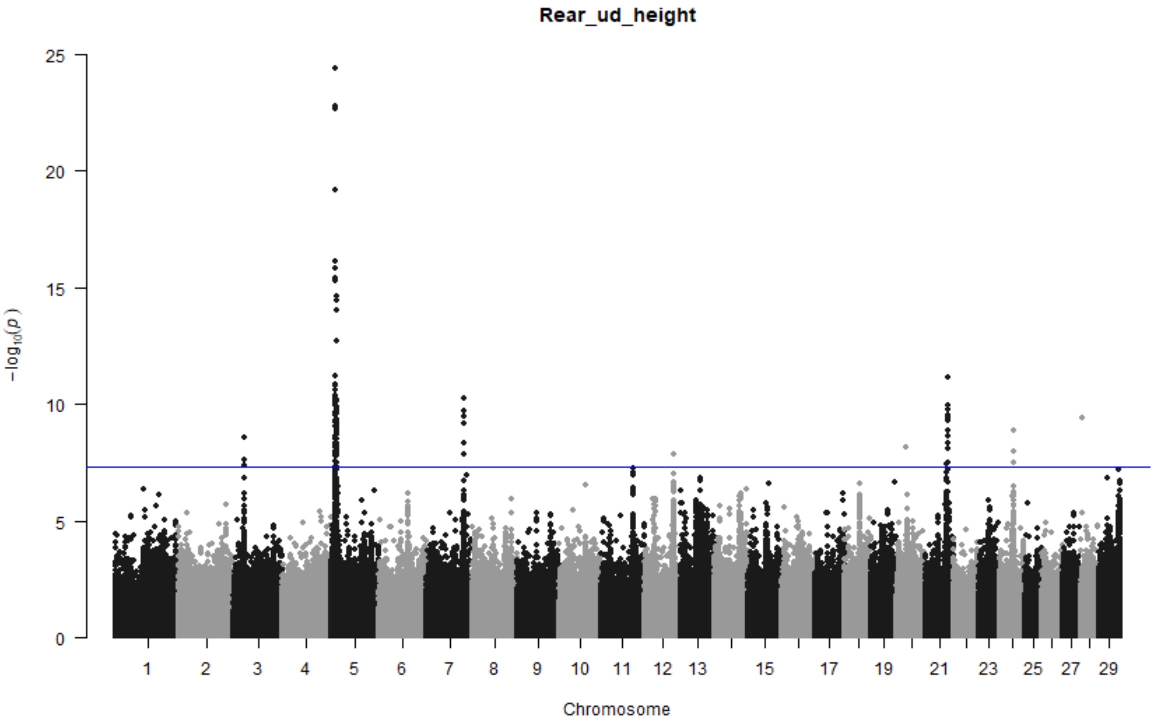
Rear_ud_height
−log10(p)
25
20
15
10
5
0
1
2
3
4
5
6
7
8
9
10
11
12
13
15
17
19
21
23
25
27
29
Chromosome

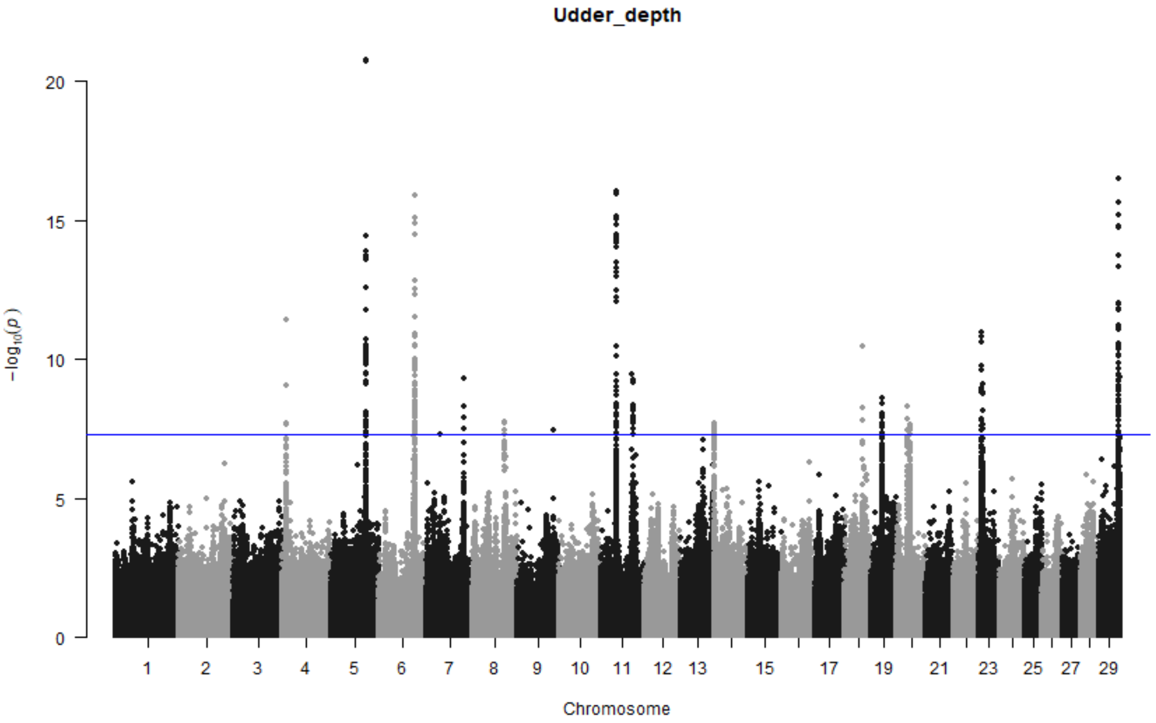
Udder_depth
20
15
10
5
0
−log10(p)
1
2
3
4
5
6
7
8
9
10
11
12
13
15
17
19
21
23
25
27
29
Chromosome

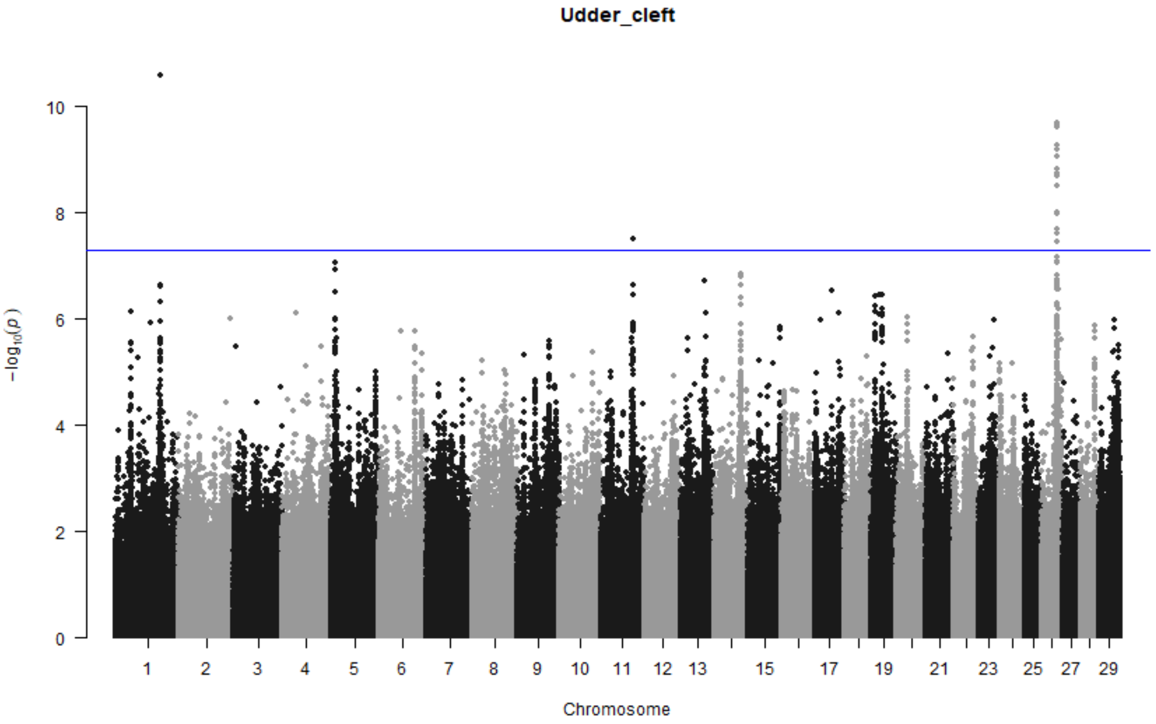
Udder_cleft
10
8
6
4
2
0
−log10(p)
1
2
3
4
5
6
7
8
9
10
11
12
13
15
17
19
21
23
25
27
29
Chromosome

Front_teat_pla

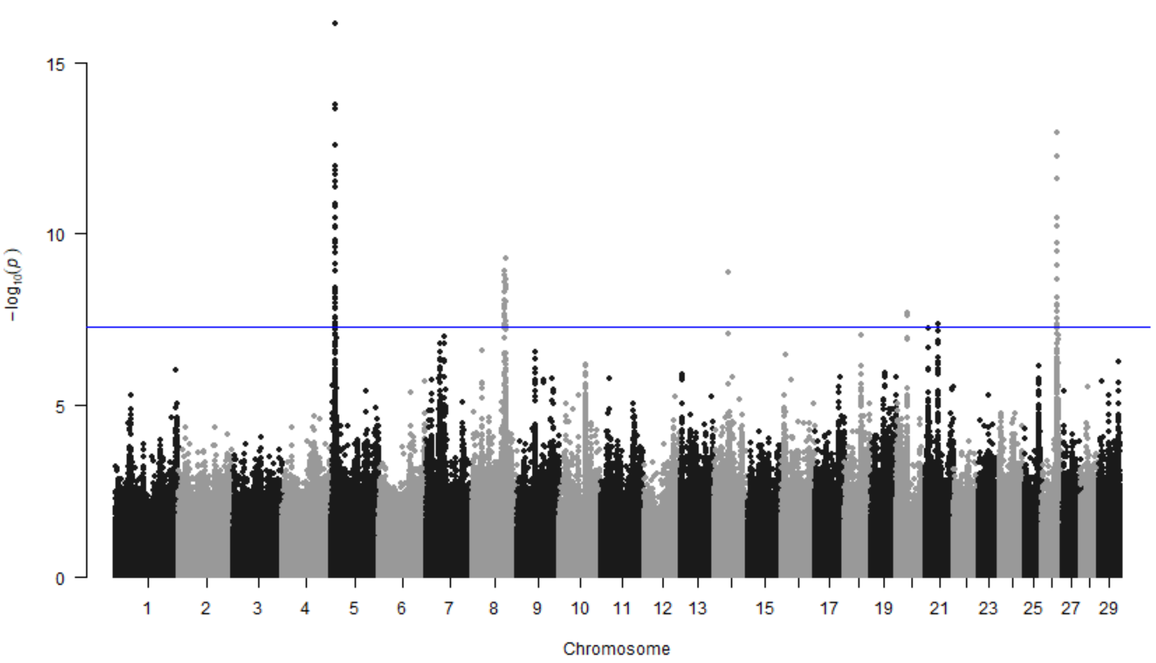

Teat_length

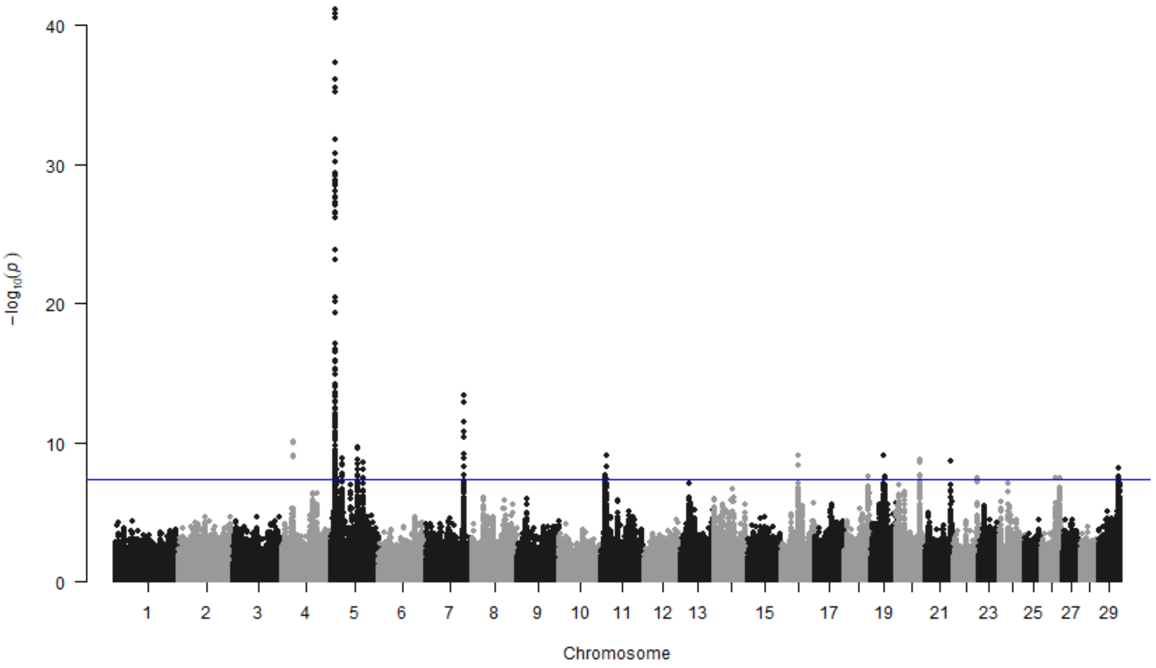

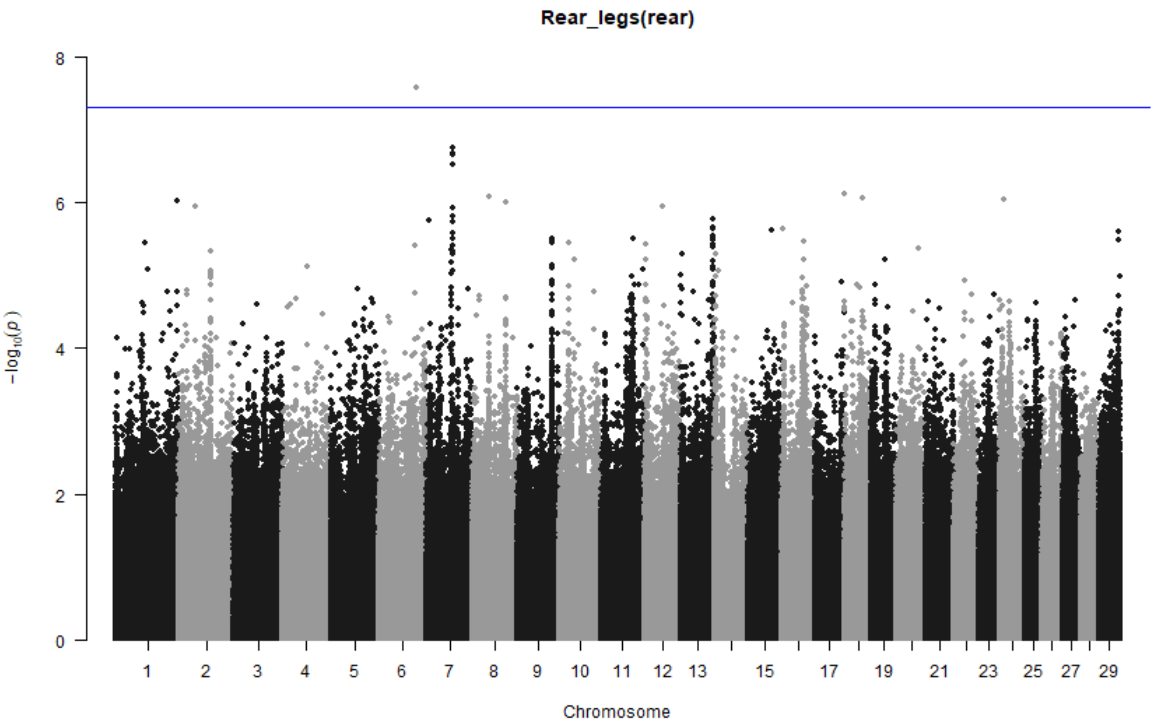
Rear_legs(rear)
8
6
4
2
0
−log10(p)
1 2 3 4 5 6 7 8 9 10 11 12 13 15 17 19 21 23 25 27 29
Chromosome

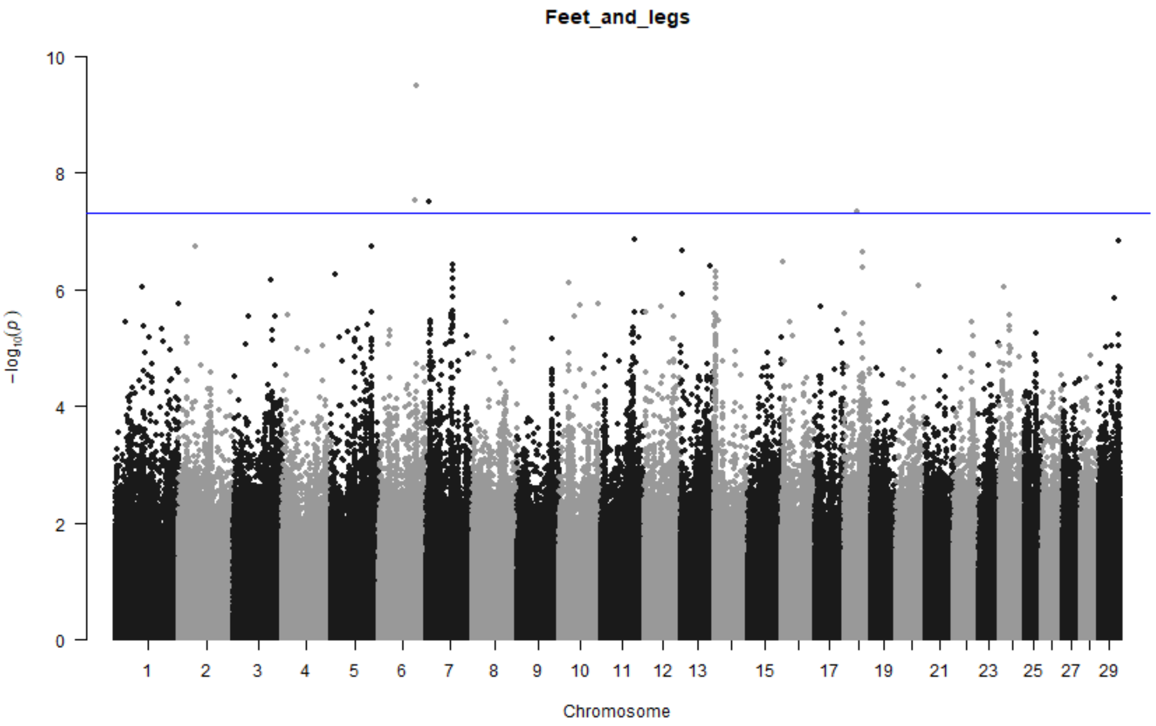
Feet_and_legs
10
8
6
4
2
0
−log10(p)
1 2 3 4 5 6 7 8 9 10 11 12 13 15 17 19 21 23 25 27 29
Chromosome

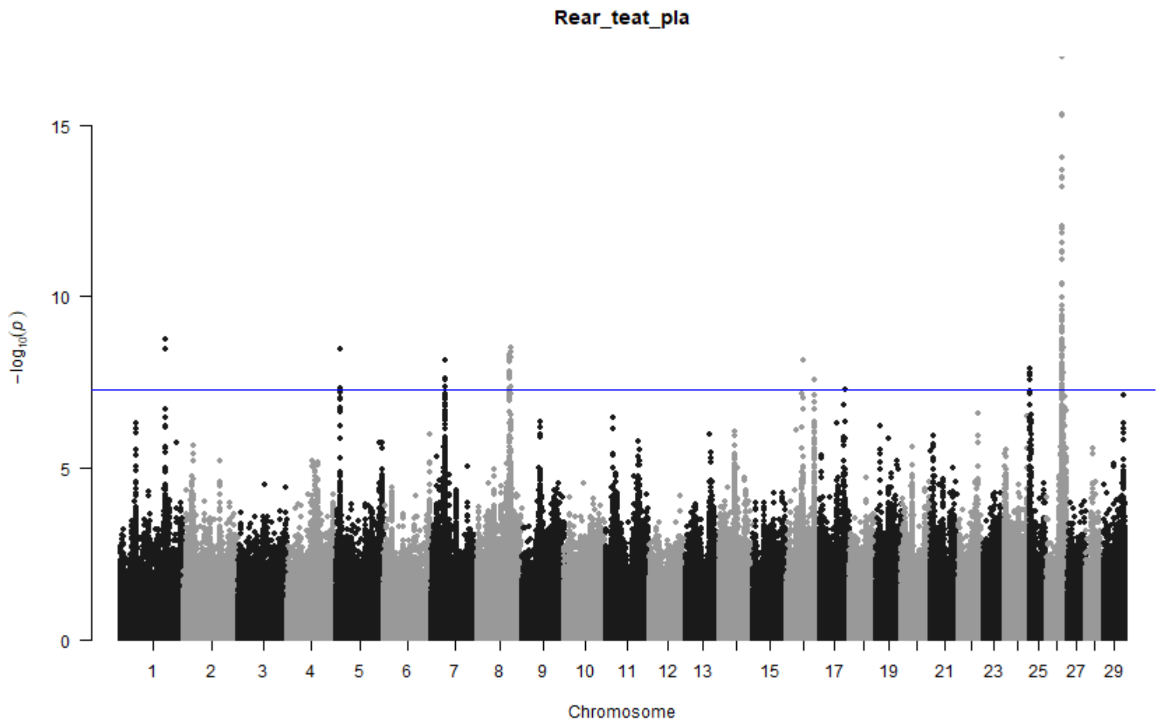

Rear_teat_pla
15
10
5
0
−log10(p)
1
2
3
4
5
6
7
8
9
10
11
12
13
15
17
19
21
23
25
27
29
Chromosome

Supplementary Figure 2. Clustering of dairy traits after removing net merit (top) and dairy form (bottom).

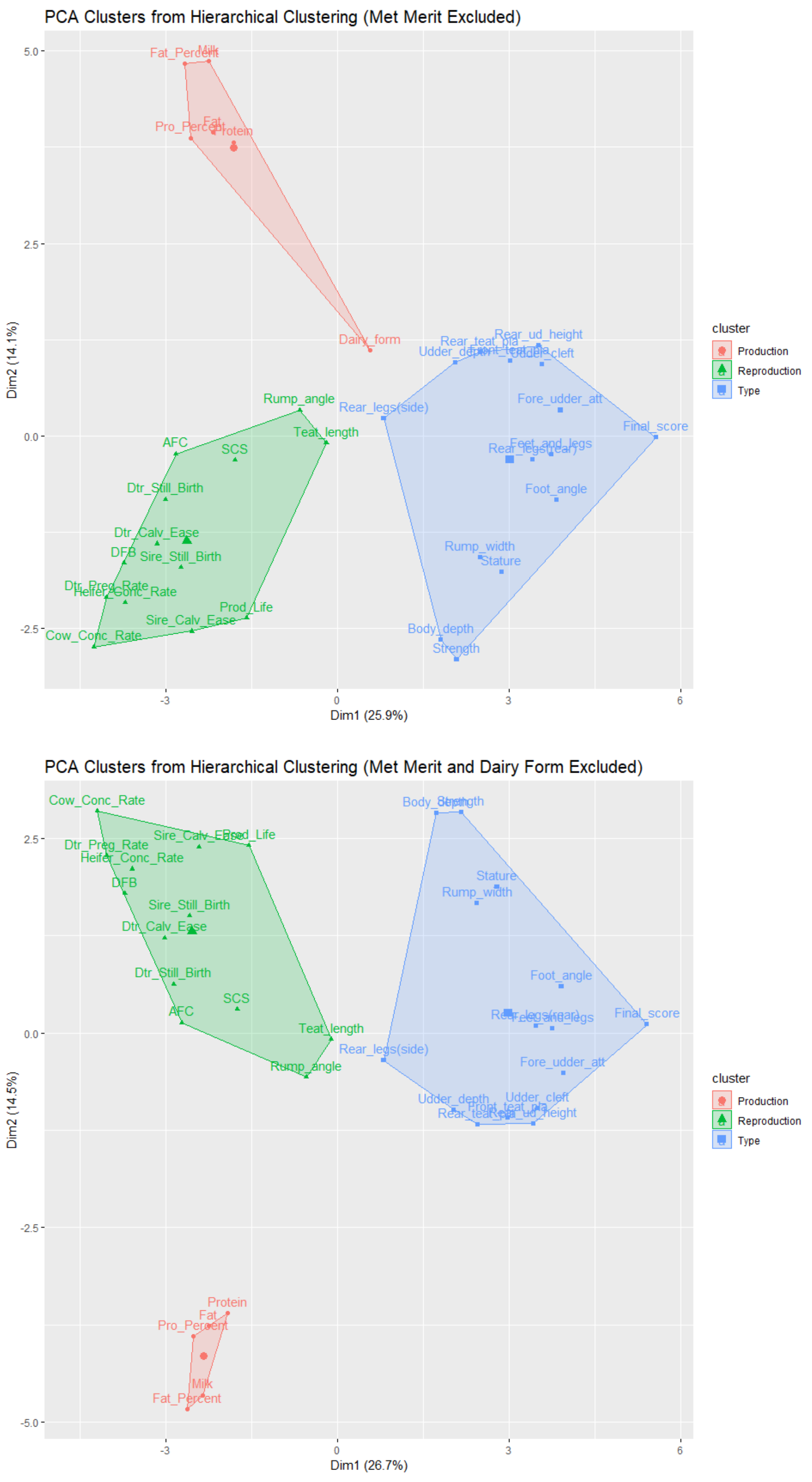

Supplementary Figure 3. Multi-trait association tests compared to single-trait association tests of five production traits on Chr12:56Mb-59Mb. As shown in the panel for protein, the lead variant in protein GWAS (indicated by the left red line) is >1 Mb away from the lead variant in multi-trait association (indicated by the right the red line). Additionally, the lead variant in multi-trait association has a much smaller *p*-value in multi-trait association than in GWAS for any individual trait.

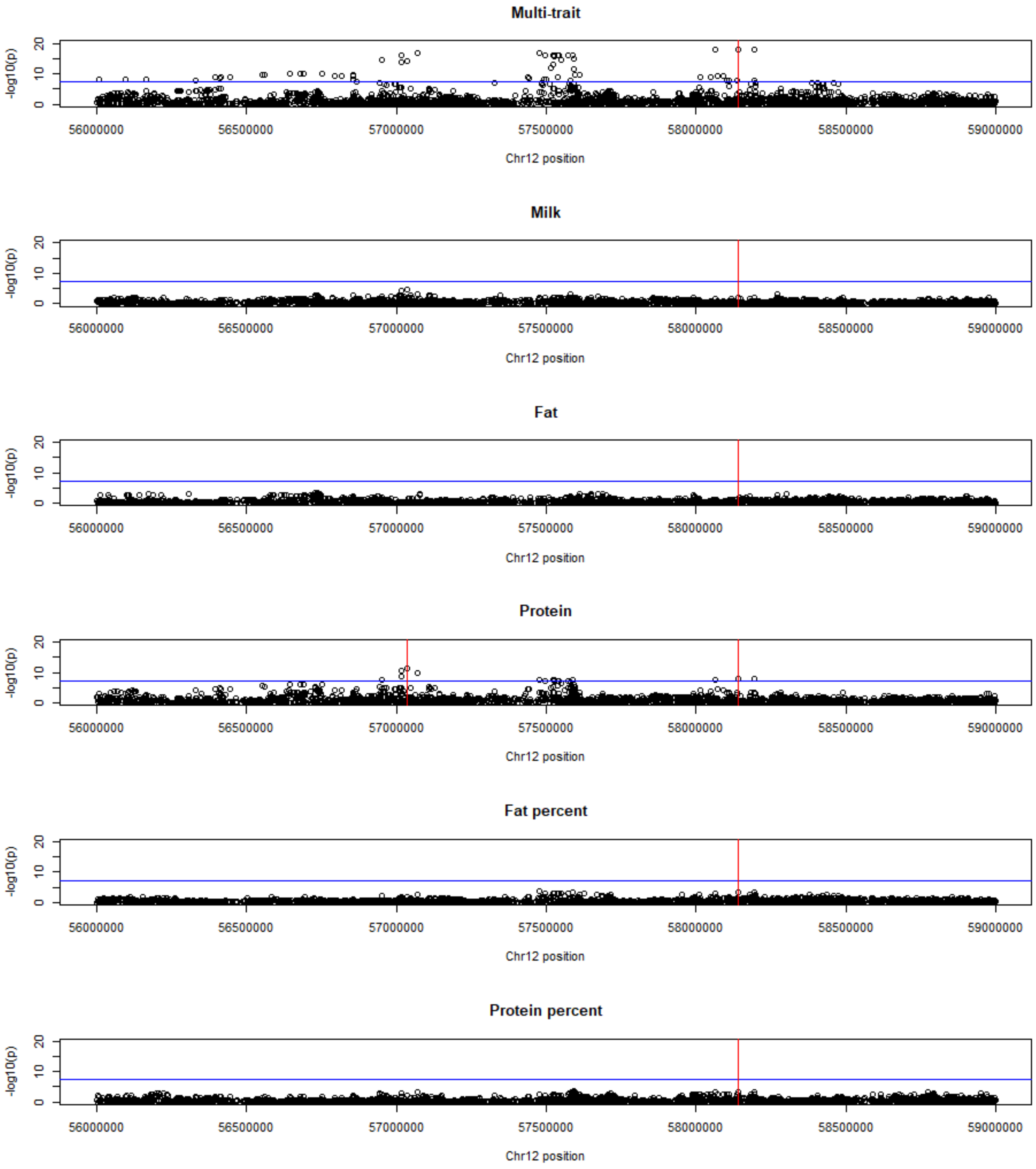

Supplementary Figure 4. Null distribution generated by permutation and actual observation for the ratio of the enrichment using only production traits to the enrichment using reproduction and type traits. Association signals were sampled 1,000 times. Actual observation is denoted by blue lines. *P*-value on the figure was calculated for one tail based on the null distribution. A. The null distribution of the ratio for moderate-impact variants. B. The null distribution of the ratio for low-impact variants. C. The null distribution of the ratio for modifier variants.

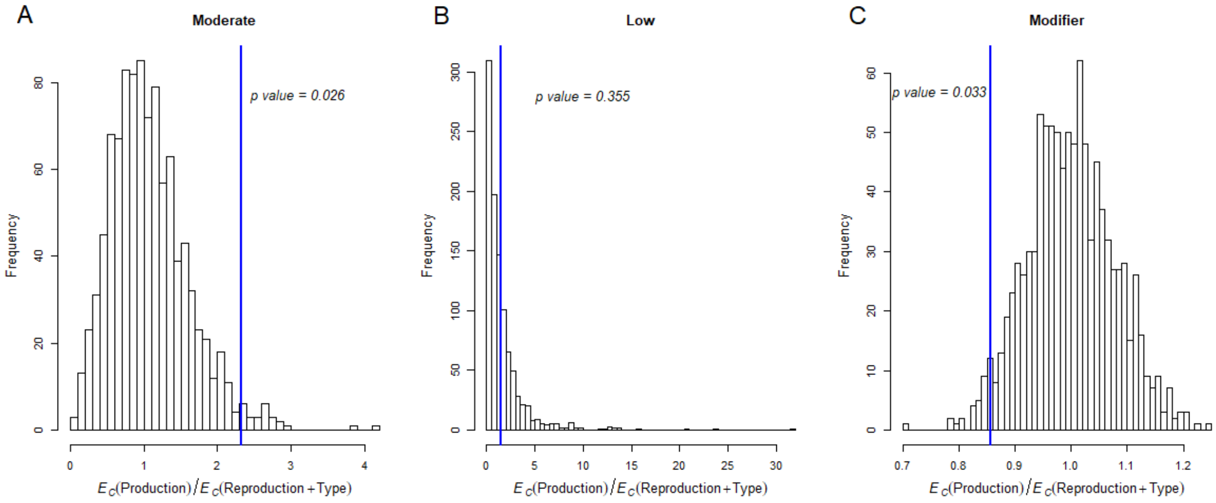

Supplementary Figure 5. Histogram of posterior probability of causality for genes.

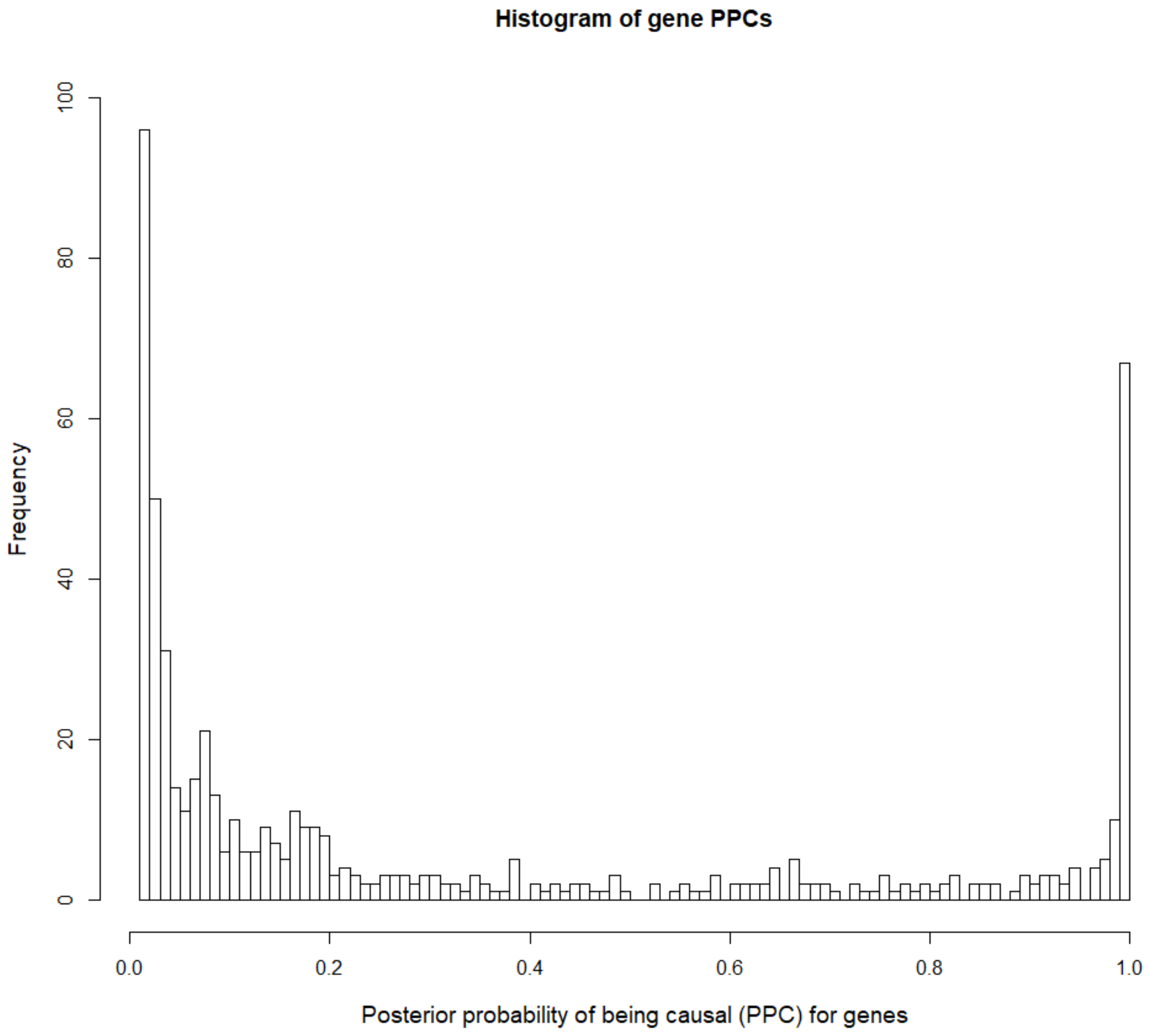

Supplementary Figure 6. Comparison between GWAS modeling reliability and GWAS not modeling reliability. A. GWAS for fat percentage with reliability modeled. B. GWAS for fat percentage without reliability modeled. C. GWAS for daughter pregnancy rate with reliability modeled. D. GWAS for daughter pregnancy rate without reliability modeled.

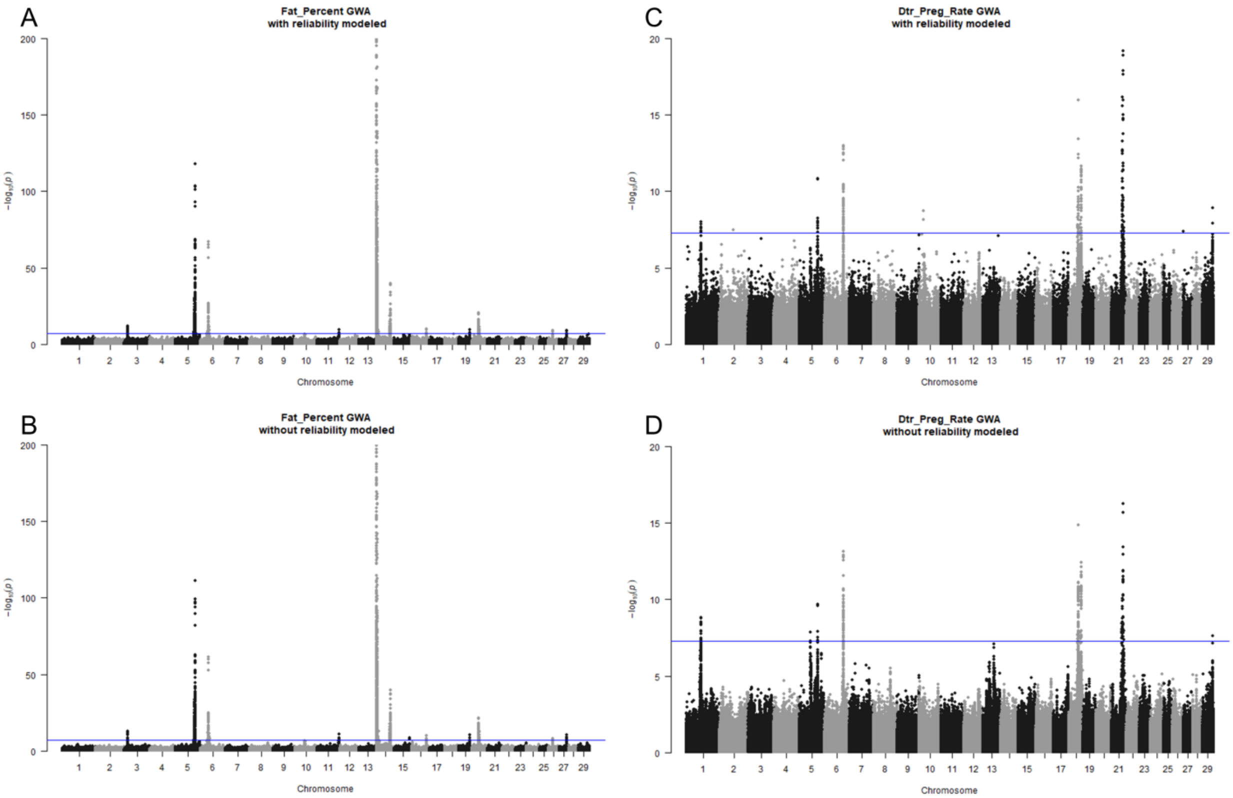

Supplementary Figure 7. Estimates of correlations between 35 dairy traits using all sequence variants and two variant subsets. A. Estimates of correlations computed with all sequence variants versus estimates of correlations computed with every 10th variant. B. Estimates of correlations computed with all sequence variants versus estimates of correlations computed with every 100th variant.

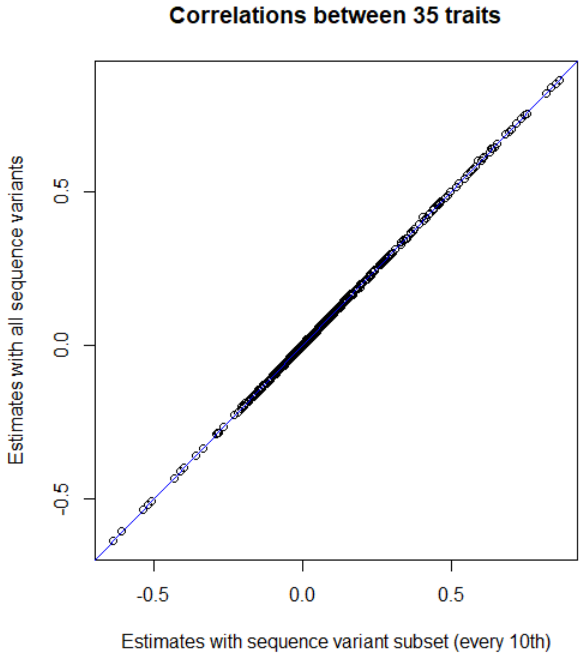

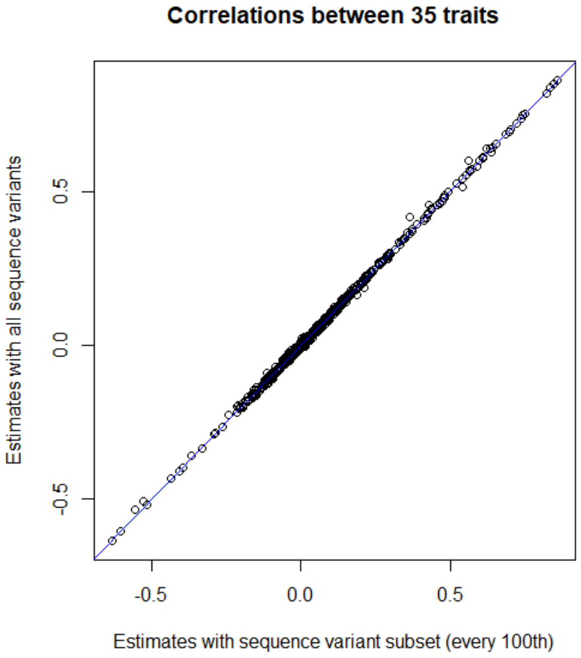

Supplementary Notes, Discussion, Methods

Part I. Trait definition

Heifer conception rate (HCR) is defined as percentage of inseminated heifers that become pregnant at each service. An HCR of 1 implies that daughters of this bull are 1% more likely to become pregnant as a heifer than daughters of a bull with an evaluation of 0.

Cow conception rate (CCR) is defined as percentage of inseminated cows that become pregnant at each service. A CCR of 1 implies that daughters of this bull are 1% more likely to become pregnant during that lactation than daughters of a bull with an evaluation of 0.

Daughter pregnancy rate (DPR) is defined as percentage of nonpregnant cows that become pregnant during each 21-day period. A DPR of 1 implies that daughters from this bull are 1% more likely to become pregnant during that estrus cycle than a bull with an evaluation of 0.
Source: https://aipl.arsusda.gov/reference/Form_GE_FFertility_1008.pdf

Daughter CE measures the ability of a particular cow (daughter) to calve easily. The CE evaluations are expressed as percent difficult births in primiparous heifers (%DBH), where difficult births are those scored as requiring considerable force or being extremely difficult (4 or 5 on a five point scale). Service Sire CE measures the tendency of calves from a particular service sire to be born more or less easily.

Daughter SB measures the ability of a particular cow (daughter) to produce live calves. Service Sire SB measures the tendency of calves from a particular service sire to be stillborn more or less often. The SB evaluations are expressed as percent stillbirths in heifers (%SBH), where stillborn calves are those scored as dead at birth or born alive but died within 48 h of birth (2 or 3 on a three point scale).
Source: https://aipl.arsusda.gov/reference/fertility/ce.htm

Part II. Computation of *P*(*D*|*M*)

$$\begin{aligned} \mathbf{y} &= \mathbf{Xb} + \mathbf{Za} + \mathbf{g} + \mathbf{e} \\ \mathbf{b} &\sim N(0, \varphi\sigma_e^2\mathbf{I}) \\ \mathbf{a} &\sim N(0, \gamma\sigma_e^2\mathbf{I}) \\ \mathbf{g} &\sim N(0, \eta\sigma_e^2\mathbf{G}) \\ \mathbf{e} &\sim N(0, \sigma_e^2\mathbf{R}) \\ P\left(\sigma_e^2\right) &\propto 1/\sigma_e^2 \end{aligned} \tag{S.1}$$

For any model M that defines a SNP set to be included in the model, let S represent the SNP set. Let $V = \varphi XX' + \gamma Z_S Z_S' + \eta G + R$, $\alpha = \frac{n}{2}$, $\beta = \frac{1}{2} y'V^{-1}y$, and $D = \{y, X, Z, G, R\}$. We have $P(D \mid M) = P\left(y \mid X, Z, G, R, M\right) P\left(X, Z, G, R \mid M\right)$. Assuming that variant genotypes alone do not contain information about model M, $P\left(X, Z, G, R \mid M\right) = P(X, Z, G, R)$ remains constant for any model M.

$$\begin{aligned} P\left(y \mid X, Z, G, R, M\right) &= \int P\left(y \mid V, \sigma_e^2\right) P(\sigma_e^2) d\sigma_e^2 \\ &= \int \left(2\pi\sigma_e^2\right)^{-\frac{n}{2}} |V|^{-\frac{1}{2}} \exp\left(-\frac{1}{2} y'V^{-1}y\sigma_e^{-2}\right) \left(\sigma_e^2\right)^{-1} d\sigma_e^2 \\ &= \frac{\Gamma(\alpha)}{\beta^\alpha} (2\pi)^{-\frac{n}{2}} |V|^{-\frac{1}{2}} \int \frac{\beta^\alpha}{\Gamma(\alpha)} \left(\sigma_e^2\right)^{-\alpha-1} \exp\left(-\frac{\beta}{\sigma_e^2}\right) d\sigma_e^2 \\ &= \frac{\Gamma(\alpha)}{\beta^\alpha} (2\pi)^{-\frac{n}{2}} |V|^{-\frac{1}{2}}. \end{aligned}$$

Thus,

$$\log P(D \mid M) \cong \log\Gamma(\alpha) - \alpha \log\beta - \frac{n}{2} \log 2\pi - \frac{1}{2} \log|V|. \tag{S.2}$$

Evaluating $\log P(D \mid M)$ involves computation of the determinant and inverse of matrix ***V***. The computations of $|V|$ and V^{-1} can be eased by applications of Sylvester's determinant identity and Woodbury matrix identity, respectively. We have $|V| = |W| \left|I + K'W^{-1}K\right|$ and $V^{-1} = W^{-1} - W^{-1}K\left(I + K'W^{-1}K\right)^{-1} K'W^{-1}$, where $W = \eta G + R$ and $K = \left[\sqrt{\varphi}X \;\; \sqrt{\gamma}Z_S\right]$. With the two equations, we only need to compute the determinant and inverse of ***W*** once and use them for

all variants or variant-sets. Inverse of $\boldsymbol{V}$ involves a matrix inversion that has a dimension equal to total number of covariates and included variants (usually much smaller than sample size *n*).

Alternatively, we can use a linear transformation to ease the evaluation of $\log P(D \mid M)$. Let $W = LL'$ and $T(\boldsymbol{v}) = \boldsymbol{L}^{-1}\boldsymbol{v}$. With the linear transformation, the model becomes

$$\begin{gathered} L^{-1}y = L^{-1}Xb + L^{-1}Za + L^{-1}(g+e) \\ y^* = X^*b + Z^*a + e^* \\ b \sim N(0, \varphi\sigma_e^2 I) \\ a \sim N(0, \gamma\sigma_e^2 I) \\ e^* \sim N(0, \sigma_e^2 I) \\ P(\sigma_e^2) \propto 1/\sigma_e^2 \end{gathered} , \qquad \text{(S.3)}$$

where $y^* = L^{-1}y$, $X^* = L^{-1}X$, and $Z^* = L^{-1}Z$. Supposing $D^* = \{y^*, X^*, Z^*\}$, we get

$$\log P(D^* \mid M) \cong P(y^* \mid X^*, Z^*, M) = \frac{\Gamma(\alpha^*)}{(\beta^*)^{\alpha^*}} (2\pi)^{-\frac{n}{2}} \left|V^*\right|^{-\frac{1}{2}},$$

where $V^* = \varphi X^* X^{*\prime} + \gamma Z^* Z^{*\prime} + I$, $\alpha^* = \frac{n}{2}$, and $\beta^* = \frac{1}{2} y^{*\prime} V^{*-1} y^*$. It is easy to show

$$P(y \mid X, Z, G, R, M) / P(y^* \mid X^*, Z^*, M) = |L|^{-1}.$$

Thus,

$$\log P(D \mid M) \cong P(y^* \mid X^*, Z^*, M) - \log|L|. \qquad \text{(S.4)}$$

Evaluating $\log P(D^* \mid M)$ requires the determinant and inverse of matrix *V**. Let $K^* = \left[\sqrt{\varphi}X^* \ \sqrt{\gamma}Z^*\right]$. Based on Sylvester's determinant identity and Woodbury matrix identity, we get $\left|V^*\right| = \left|I + K^{*\prime}K^*\right|$ and $V^{*-1} = I - K^*\left(I + K^{*\prime}K^*\right)^{-1} K^{*\prime}$, respectively.

In proper software implementation, use of equation (S.4) does not necessarily increase speed or reduce memory usage compared to direct use of equation (S.2). However, the linear transformation of model (S.1) to model (S.3) illustrates how we can calculate scaled Bayes factor with model (S.1) and get its null distribution [1].

Part III. Null distribution of Bayes factors

Zhou and Guan [1] studied the null distribution of Bayes factor (H_0: $\boldsymbol{a}=\mathbf{0}$) in a linear regression model like model (S.3). Their theory holds true as long as $\varphi \to \infty$ in model (S.3). For M_1 and the null model M_0, we can obtain

$$\begin{aligned}\log BF_D &= \log P(y|X,Z,G,R,M_1) - \log P(y|X,Z,G,R,M_0)\\ \log BF_{D^*} &= \log P(y^*|X^*,Z^*,M_1) - \log P(y^*|X^*,Z^*,M_0)\end{aligned}.$$

For any M, we have proved

$$\log P(y|X,Z,G,R,M) = \log P(y^*|X^*,Z^*,M) - \log|L|.$$

Thus, for any D corresponding to D^*,

$$BF_D = BF_{D^*}. \tag{S.5}$$

Because any D is uniquely mapped to D^* and vice versa, BF_D must have the same null distribution as BF_{D^*}. This can be illustrated by using permutation of $\boldsymbol{Z}$ (switching animal labels) to create their null distributions. Any permuted D (D_p) is uniquely mapped a permuted D^* (D_p^*) and vice versa, and $BF_{D_p} = BF_{D_p^*}$. Thus, BF_D has the same null distribution as BF_{D^*}.

We need to set $\varphi \to \infty$ in model (S.3) to apply the theory of Zhou and Guan [1]. In practice, a large value (e.g., 1E8) suffices.

Define $P = I - X^*\left(X^{*\prime}X^*\right)^{-1}X^{*\prime}$, and $T = PZ^*$.

$H \overset{\text{def}}{=} T\left(T'T + \gamma^{-1}I\right)^{-1}T'$.

Let $\left(\lambda_1,\cdots,\lambda_p\right)$ be eigenvalues of H.

$$2\log \text{BF} = \sum_{i=1}^{p}\lambda_i Q_i + \sum_{i=1}^{p}\log\left(1-\lambda_i\right) \text{ with } Q_i \sim \chi_1^2.$$

So, p-value for $\log \text{BF}$ (H_0: $\boldsymbol{a}=\mathbf{0}$) can be computed by evaluating a weighted sum of chi-squared random variables. We can also compute scaled Bayes factors (sBF) [1]:

$$2\log \text{sBF} \overset{\text{def}}{=} 2\log \text{BF} - \text{E}_0\left(2\log \text{BF}\right) = 2\log \text{BF} - \sum_{i=1}^{p}\lambda_i - \sum_{i=1}^{p}\log\left(1-\lambda_i\right).$$

Part IV. Bayes factor and *p*-value in fine-mapping

When existing covariates (including variants that have been added) have an infinite value for φ (φ=1E8 suffices) and design matrix X*, adding variant i with transformed genotypes Z_i^* results in:

$$2\log \mathrm{BF} = \lambda_i Q - \log\left(1 + T_i' T_i \gamma\right)$$

$$\text{with } P = I - X^*\left(X^{*\prime} X^*\right)^{-1} X^{*\prime},\ T_i = PZ_i^*,\ \lambda_i = \frac{T_i' T_i}{T_i' T_i + 1/\gamma} \text{ and } Q \sim \chi_1^2.$$

In practice, we can first compute $\log \mathrm{BF}$ and $\mathrm{E}_0[\log \mathrm{BF}]$, and then compute $\log \mathrm{sBF}$:

$$2\log \mathrm{sBF} = 2\log \mathrm{BF} - \mathrm{E}_0\left[2\log \mathrm{BF}\right] = \lambda_i\left(Q - 1\right)$$

$$\text{with } \mathrm{E}_0\left[2\log \mathrm{BF}\right] = \lambda_i - \log\left(1 + T_i' T_i \gamma\right).$$

We set $\gamma = 1E8$ for all variants in fine-mapping, so we get $\lambda_i = 1$ for any variant i. Therefore, *p*-value can be easily computed because we have $\left(2\log \mathrm{sBF} + 1\right) \sim \chi_1^2$.

References

1. Zhou Q, Guan Y. On the Null Distribution of Bayes Factors in Linear Regression. *Journal of the American Statistical Association* 113.523, 1362-1371 (2018).
