# Supplementary material for: Functional annotation and Bayesian fine-mapping reveals candidate genes for important agronomic traits in Holstein bulls
Source: Commun Biol. 2019 Jun 18;2:212. doi: 10.1038/s42003-019-0454-y (PMC6582147; doi:10.1038/s42003-019-0454-y)
Supplement: Supplementary file 2 — Description of Additional Supplementary Files [file 42003_2019_454_MOESM2_ESM.docx]

Supplementary Data 1. Genomic control factor and number of QTL of single-trait GWAS for 35 dairy traits.

Supplementary Data 2. QTLs from single-trait GWAS of 35 dairy cattle traits.

Supplementary Data 3. Candidate regions identified by multi-trait association tests compared to those identified by single-trait GWAS.

Supplementary Data 4. The list of candidate regions identified by multi-trait association tests but missed by single-trait GWAS.

Supplementary Data 5. Number of fine-mapped signals for each trait-region association pair after filtering by a significance threshold of 5*E*-7.

Supplementary Data 6. All fine-mapping results.

Supplementary Data 7. Enrichment estimates for location of variants regarding protein-coding genes.

Supplementary Data 8. Enrichment estimates for SnpEff effect impact.

Supplementary Data 9. Enrichment estimates for GERP constrained elements.

Supplementary Data 10. Enrichment estimates for effect impact by GERP constrained elements.

Supplementary Data 11. Enrichment estimates for effect impact generated using three groups of traits.

Supplementary Data 12. All genes fine-mapped with posterior probability of causality of >0.01.

Supplementary Data 13. Variants merged from 95% credible variant sets of all fine-mapped association signals.

Supplementary Data 14. QTLdb traits corresponding to the 35 traits in this study.

Supplementary Data 15. Differences between BFMAP and the fine-mapping approach by Huang et al.

Supplementary Data 16. Genomic regions for fine-mapping.

Supplementary Data 17. Number of variants and effective number of independent variants in genomic regions for fine-mapping.
