# Supplementary material for: Functional annotation and Bayesian fine-mapping reveals candidate genes for important agronomic traits in Holstein bulls
Source: Commun Biol. 2019 Jun 18;2:212. doi: 10.1038/s42003-019-0454-y (PMC6582147; doi:10.1038/s42003-019-0454-y)
Supplement: Supplementary file 3 — Reporting Summary [file 42003_2019_454_MOESM3_ESM.pdf]

nature research

Corresponding author(s): Li Ma

Reporting Summary

Nature Research wishes to improve the reproducibility of the work that we publish. This form provides structure for consistency and transparency in reporting. For further information on Nature Research policies, see Authors & Referees and the Editorial Policy Checklist.

Statistical parameters

When statistical analyses are reported, confirm that the following items are present in the relevant location (e.g. figure legend, table legend, main text, or Methods section).

n/a	Confirmed	
☐	☒	The exact sample size (n) for each experimental group/condition, given as a discrete number and unit of measurement
☐	☒	An indication of whether measurements were taken from distinct samples or whether the same sample was measured repeatedly
☐	☒	The statistical test(s) used AND whether they are one- or two-sided<br>*Only common tests should be described solely by name; describe more complex techniques in the Methods section.*
☐	☒	A description of all covariates tested
☐	☒	A description of any assumptions or corrections, such as tests of normality and adjustment for multiple comparisons
☐	☒	A full description of the statistics including central tendency (e.g. means) or other basic estimates (e.g. regression coefficient) AND variation (e.g. standard deviation) or associated estimates of uncertainty (e.g. confidence intervals)
☐	☒	For null hypothesis testing, the test statistic (e.g. F, t, r) with confidence intervals, effect sizes, degrees of freedom and P value noted<br>*Give P values as exact values whenever suitable.*
☐	☒	For Bayesian analysis, information on the choice of priors and Markov chain Monte Carlo settings
☐	☒	For hierarchical and complex designs, identification of the appropriate level for tests and full reporting of outcomes
☐	☒	Estimates of effect sizes (e.g. Cohen's d, Pearson's r), indicating how they were calculated
☐	☒	Clearly defined error bars<br>*State explicitly what error bars represent (e.g. SD, SE, CI)*

Our web collection on statistics for biologists may be useful.

Software and code

Policy information about availability of computer code

Data collection	Cattle constrained elements: ftp://ftp.ensembl.org/pub/release-90/bed/ensembl-compara/68_eutherian_mammals_gerp_constrained_elements/gerp_constrained_elements.bos_taurus.bed.gz<br>Cattle genome annotation: ftp://ftp.ncbi.nlm.nih.gov/genomes/all/GCF_000003055.6_Bos_taurus_UMD_3.1.1/GCF_000003055.6_Bos_taurus_UMD_3.1.1_genomic.gff.gz<br>Cattle QTLdb: https://www.animalgenome.org/cgi-bin/QTLdb/BT/index<br>Cattle genome variation: ftp://ftp.ensembl.org/pub/release-89/variation/gvf/bos_taurus/
Data analysis	BFMAP: https://jiang18.github.io/bfmap/<br>MMAP: https://mmap.github.io/

For manuscripts utilizing custom algorithms or software that are central to the research but not yet described in published literature, software must be made available to editors/reviewers upon request. We strongly encourage code deposition in a community repository (e.g. GitHub). See the Nature Research guidelines for submitting code & software for further information.

Data

Policy information about availability of data

All manuscripts must include a data availability statement. This statement should provide the following information, where applicable:

- Accession codes, unique identifiers, or web links for publicly available datasets
- A list of figures that have associated raw data
- A description of any restrictions on data availability

The computing program is available at https://jiang18.github.io/bfmap/. The original genotype data are owned by third parties and maintained by the Council on Dairy Cattle Breeding (CDCB). A request to CDCB is necessary for getting data access on research, which may be sent to: João Dürr, CDCB Chief Executive Officer (joao.durr@cdcb.us). All other data have been shown in the manuscript and supplementary data. All other relevant data are available in the manuscript, Supporting Information files, and from the corresponding author upon request.

Field-specific reporting

Please select the best fit for your research. If you are not sure, read the appropriate sections before making your selection.

☒ Life sciences ☐ Behavioural & social sciences ☐ Ecological, evolutionary & environmental sciences

For a reference copy of the document with all sections, see nature.com/authors/policies/ReportingSummary-flat.pdf

Life sciences study design

All studies must disclose on these points even when the disclosure is negative.

Sample size	This paper focused on a new method with application to real data. The study included over 27,000 dairy bulls with highly reliable phenotype that should provide adequate power.
Data exclusions	A set of quality control edits were applied to the data. Details have been included in the Method section.
Replication	Not applicable.
Randomization	Not applicable because our study is an observational study.
Blinding	NA

Reporting for specific materials, systems and methods

Materials & experimental systems

n/a	Involved in the study
☒	☐ Unique biological materials
☒	☐ Antibodies
☒	☐ Eukaryotic cell lines
☒	☐ Palaeontology
☒	☐ Animals and other organisms
☒	☐ Human research participants

Methods

n/a	Involved in the study
☒	☐ ChIP-seq
☒	☐ Flow cytometry
☒	☐ MRI-based neuroimaging
